# Development of a cardiovascular magnetic resonance‐compatible large animal isolated heart model for direct comparison of beating and arrested hearts

**DOI:** 10.1002/nbm.4692

**Published:** 2022-02-12

**Authors:** Andrew D. Scott, Tim Jackson, Zohya Khalique, Margarita Gorodezky, Ben Pardoe, Lale Begum, V. Domenico Bruno, Rasheda A. Chowdhury, Pedro F. Ferreira, Sonia Nielles‐Vallespin, Malte Roehl, Karen P. McCarthy, Padmini Sarathchandra, Jan N. Rose, Denis J. Doorly, Dudley J. Pennell, Raimondo Ascione, Ranil de Silva, David N. Firmin

**Affiliations:** ^1^ Cardiovascular Magnetic Resonance Unit Royal Brompton Hospital London UK; ^2^ National Heart and Lung Institute Imperial College London UK; ^3^ Department of Perfusion Royal Brompton Hospital London UK; ^4^ Translational Biomedical Research Centre University of Bristol Bristol UK; ^5^ Bristol Heart Institute University Hospital Bristol NHS Foundation Trust Bristol UK; ^6^ Imperial Centre for Cardiac Engineering Imperial College London UK; ^7^ Cardiac Morphology Unit Royal Brompton Hospital London UK; ^8^ Magdi Yacoub Institute, National Heart and Lung Institute Imperial College London UK; ^9^ Department of Aeronautics Imperial College London UK

**Keywords:** cardiovascular magnetic resonance, diffusion tensor imaging, Langendorff perfusion, microstructure, myocardial tissue characterization, oedema, preclinical

## Abstract

Cardiac motion results in image artefacts and quantification errors in many cardiovascular magnetic resonance (CMR) techniques, including microstructural assessment using diffusion tensor cardiovascular magnetic resonance (DT‐CMR). Here, we develop a CMR‐compatible isolated perfused porcine heart model that allows comparison of data obtained in beating and arrested states. Ten porcine hearts (8/10 for protocol optimisation) were harvested using a donor heart retrieval protocol and transported to the remote CMR facility. Langendorff perfusion in a 3D‐printed chamber and perfusion circuit re‐established contraction. Hearts were imaged using cine, parametric mapping and STEAM DT‐CMR at cardiac phases with the minimum and maximum wall thickness. High potassium and lithium perfusates were then used to arrest the heart in a slack and contracted state, respectively. Imaging was repeated in both arrested states. After imaging, tissue was removed for subsequent histology in a location matched to the DT‐CMR data using fiducial markers. Regular sustained contraction was successfully established in six out of 10 hearts, including the final five hearts. Imaging was performed in four hearts and one underwent the full protocol, including colocalised histology. The image quality was good and there was good agreement between DT‐CMR data in equivalent beating and arrested states. Despite the use of autologous blood and dextran within the perfusate, T2 mapping results, DT‐CMR measures and an increase in mass were consistent with development of myocardial oedema, resulting in failure to achieve a true diastolic‐like state. A contiguous stack of 313 5‐μm histological sections at and a 100‐μm thick section showing cell morphology on 3D fluorescent confocal microscopy colocalised to DT‐CMR data were obtained. A CMR‐compatible isolated perfused beating heart setup for large animal hearts allows direct comparisons of beating and arrested heart data with subsequent colocalised histology, without the need for onsite preclinical facilities.

## Graphical abstract

A cardiovascular magnetic resonance (CMR)‐compatible isolated perfused beating porcine heart model was developed that allows direct comparison of data acquired in multiple states of contraction between the beating and arrested heart. We describe the development of this model, its successes and failures, including the presence of oedema in the hearts. In this study, microstructural measures were obtained from the porcine hearts using diffusion tensor CMR in the most and least contracted states with subsequent colocalised histology.

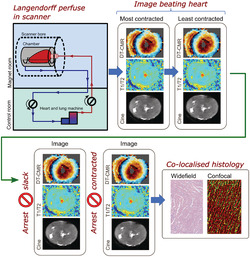

**Abbreviations used**
bSSFP: balanced steady state free precession
CMR: cardiovascular magnetic resonance
DAPI: 4′,6‐diamidino‐2‐phenylindole
DT‐CMR: diffusion tensor cardiovascular magnetic resonance
E2A: absolute angulation of the second eigenvector, a measure of sheetlet orientation
EPI: echo planar imaging
FA: fractional anisotropy
HA: helix angle
LV: left ventricle
MD: mean diffusivity
MOLLI: modified Look Locker imaging
SENSE: sensitivity encoding
STEAM: stimulated echo acquisition mode
TBRC: Translational Biomedical Research Centre, University of Bristol
WGA: wheat germ agglutinin

## 1 INTRODUCTION

Despite several decades of development, many cardiovascular magnetic resonance (CMR) methods are hampered by the effects of cardiac motion.^1^ In diffusion tensor CMR (DT‐CMR), measurement of the diffusion of water molecules on a scale of 10s of μm, while the heart moves on a scale approximately three orders of magnitude larger, is challenging. In vivo DT‐CMR provides an unparalleled insight into the myocardial microstructure and its dynamics,^2^, ^3^ with clinical studies demonstrating novel insights into cardiomyopathies, congenital heart disease and amyloidosis.^4^, ^5^, ^6^, ^7^ One widely adopted and validated method for assessing the dynamic microstructure is the stimulated echo acquisition mode (STEAM) sequence that divides diffusion encoding between two consecutive cardiac cycles.^4^, ^8^, ^9^, ^10^ This allows the diffusion‐encoding gradients to be short relative to cardiac motion, with a long time between the gradients to enable the water molecules to diffuse relatively long distances and thus provide measurable diffusion‐related signal loss with minimal artefact caused by bulk motion. However, the cyclical strain of the heart during the time that the water molecules are diffusing (the diffusion time, ∆) results in augmentation of the measured diffusivity, despite the fact that the heart is in an identical position and state when both diffusion‐encoding gradients are applied.^11^ This strain effect is due to the compression of distances diffused in a stretched medium once the medium returns to its original state (or vice versa for a compressed medium). Recent work has shown that this strain effect is considerably overcorrected by existing models, which assume a jelly‐like myocardium,^12^ however, the precise magnitude of the effect of strain on in vivo STEAM DT‐CMR parameters is unknown. Comparisons between a beating and arrested in situ or ex vivo heart in the same animal have gone some way to providing solutions,^4^, ^13^ but the arrested heart in each animal can only be arrested in either a systolic‐ or diastolic‐like state and the arrested heart is unloaded. Precise quantification of the effect of strain on the DT‐CMR results is vital for ensuring that future clinical studies are able to correctly differentiate the measured changes to pathological alterations in microstructure and those due to changes in myocardial strain.

A number of methodologies have been developed to study the effects of motion on CMR data including DT‐CMR, for example, numerical simulations,^14^ dynamic phantoms^15^ and comparing data acquired in vivo with equivalent results obtained ex vivo or in situ after cardiac arrest.^4^, ^16^ However, simulations are inherently simplistic and phantoms are difficult to construct with realistic cardiac‐like motion. Comparisons of beating in vivo with arrested in situ hearts may be more realistic, but do mean that the effects of cardiac motion cannot be distinguished from the effects of respiratory motion and/or breath‐holding on the data.

Langendorff perfusion is a well‐established method for maintaining a beating isolated perfused heart ex vivo.^17^ Isolated perfused hearts offer several benefits over in vivo animal models, including high coil fill factors and potentially controlled ventricular loading. The use of isolated hearts within an MRI scanner is, however, complicated by the strong static and time‐varying magnetic fields inherent to the technique: ferromagnetic objects such as the pump used to perfuse the heart are potential projectiles, induced currents in conductors result in heating, and sharp changes in magnetic susceptibility cause image artefacts. Despite this, a number of studies have demonstrated large and small mammalian isolated beating heart models within an MRI scanner.^18^, ^19^ In this work we describe the development of a large animal MRI‐compatible isolated perfused heart model. While previous work^20^ used Langendorff perfusion to arrest rat hearts in multiple states of contraction, we aim to allow direct comparison of equivalent beating and arrested states with subsequent colocalised histology. Such a model would allow precise quantitation of the effects of cardiac motion on CMR methods, including the effects of strain on in vivo STEAM DT‐CMR measures. We also aim to demonstrate that isolated perfused hearts facilitate geographical separation of the preclinical and imaging facilities, allowing collaboration between specialists in both areas. We fully describe our developmental experience to provide others with a guide when developing similar models.

## 2 EXPERIMENTAL

**FIGURE 1:**
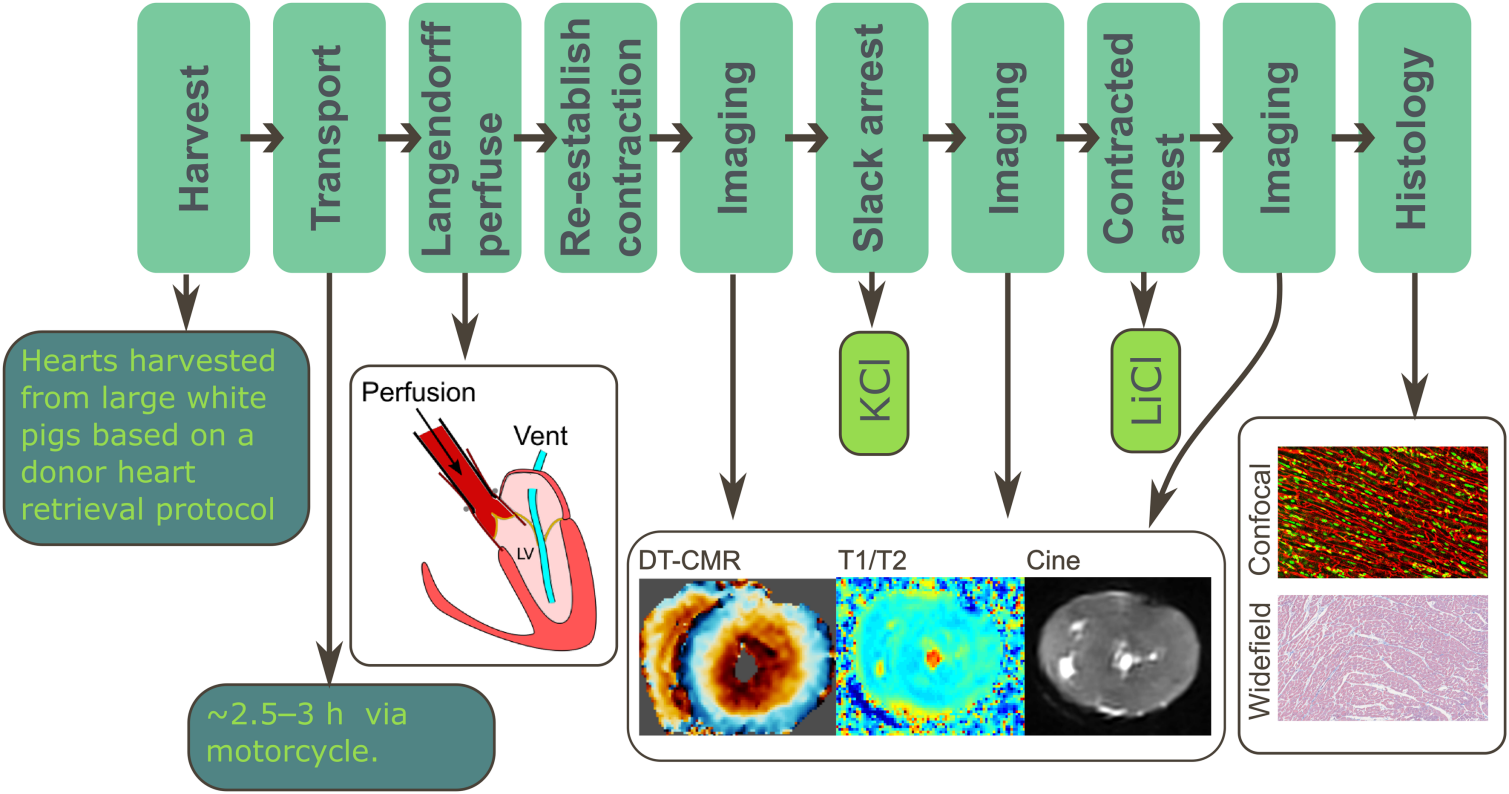
A schematic of the protocol design. A full description of the experimental protocol is provided in the text. See subsequent figures for colour‐bars, units and the description of histology

While techniques were refined in the course of our work, we describe the final experimental protocol schematically in Figure 1 and below. Porcine hearts were explanted in a preclinical facility and then transported to the imaging facility. The hearts were Langendorff‐perfused to re‐establish contraction and then imaged while beating. The perfusate was then switched to another formulation to induce arrest in a slack state before repeating the imaging step. Switching the perfusate again induced contracture and imaging was repeated a third time. Tissue blocks were removed for subsequent histological assessment.

### 2.1 Heart harvest

To avoid keeping lone animals, two hearts were harvested at the Translational Biomedical Research Centre (TBRC), University of Bristol, a Good Laboratory Practice Monitoring Authority standard (UK) preclinical facility, on each day of experiments conducted. Relevant licences were provided by the UK Home Office. In total, 10 hearts were harvested from large white pigs (60–70 kg) based on a donor heart retrieval protocol. Eight hearts were used in protocol optimisation, the protocol up to and including imaging at slack arrest was achieved in two hearts and the full protocol was performed in one heart. Animals were anaesthetised and mechanically ventilated by senior veterinary anaesthetists with ketamine, midazolam, dexmedetomidine and propofol as necessary. Procedures were performed by senior cardiac surgeons with full monitoring of key vital parameters during surgery. One to two units (300–600 ml) of autologous blood was harvested from each animal at clinical standards and stored in heparinised blood storage bags at 4°C. After median sternotomy, heparin was administered to achieve an activated coagulation time of longer than 250 s. Next, the superior and inferior vena cava and the aorta were clamped in sequence and 500 ml of cold (4°C) cardioplegia (St Thomas' solution) was administered into the aortic root at high pressure (>200 mmHg) to achieve standstill (Video S1). The arrested hearts were promptly removed from the chest, with a section of aorta up to the level of the first branch vessel intact. The individual hearts were placed in plastic containers filled with cold cardioplegia, which were then placed inside bags filled with crushed ice. Hearts and blood were placed within insulated boxes filled with more crushed ice. Hearts were transported individually immediately after harvest ~210 km to the imaging centre (Royal Brompton Hospital, London) via motorcycle courier, resulting in a cold ischaemic time of ~2.5–3 h.

### 2.2 Experimental setup

**FIGURE 2:**
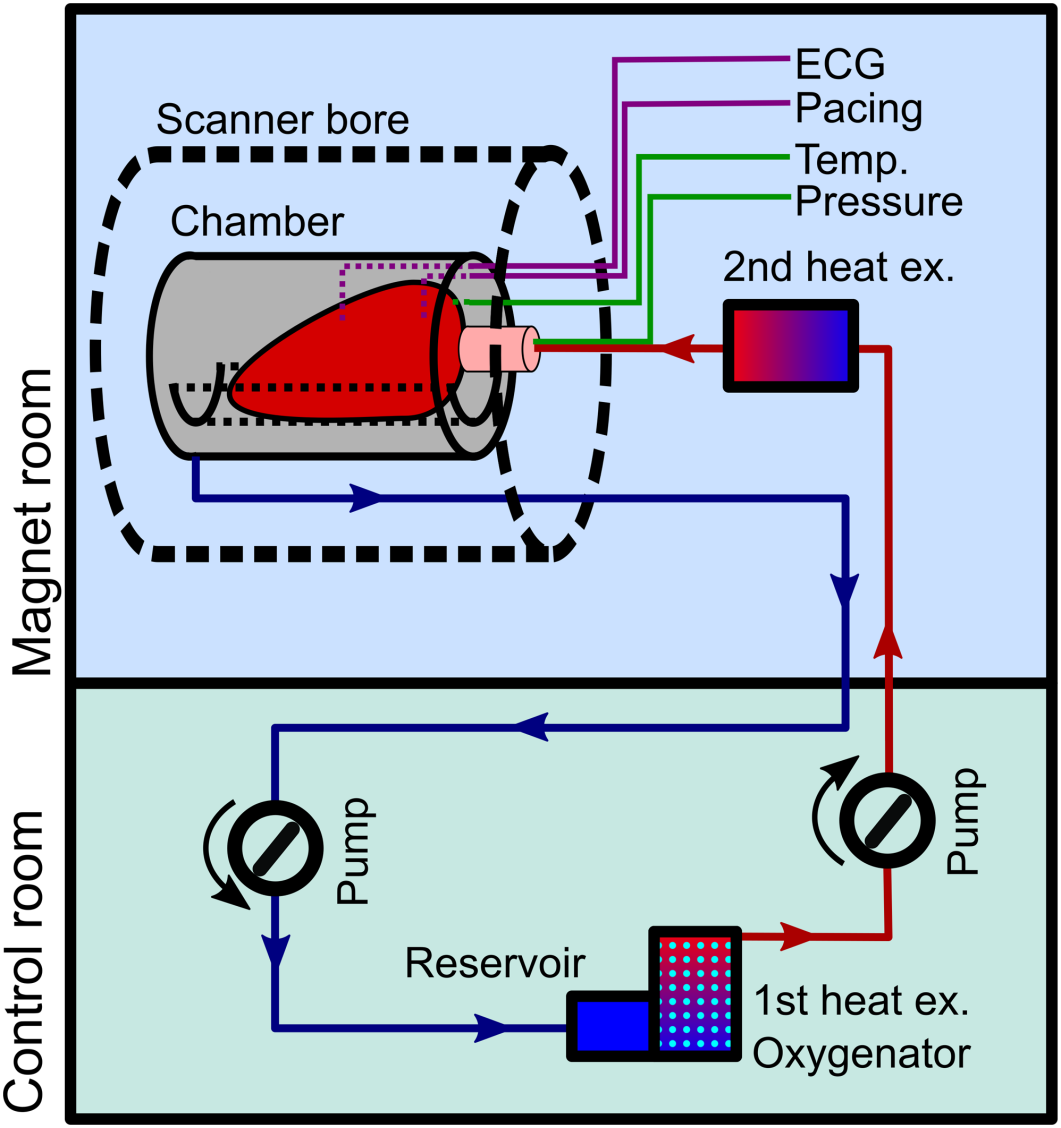
A schematic of the experimental setup. Long tubing lines connected the heat exchanger/oxygenator (which also served as a reservoir) located in the control room to the heart within the chamber at the magnet. A second heat exchanger, located on the bed of the scanner, accounted for heat loss in the perfusion tubing. Pacing, ECG and temperature sensors entered the chamber via ports and perfusion pressure was measured in the aorta cannula near the coronary ostia

Hearts were Langendorff‐perfused using a standard clinical heart and lung machine and a paediatric hollow fibre membrane oxygenator, heat exchanger and reservoir (Dideco Kids D101, LivaNova Mirandola, Italy). A custom perfusion circuit (Figure 2) was developed using standard PVC perfusion tubing (Extra Soft, Sorin Group, London, UK). Long perfusion tubing lines (6.25 mm outer diameter, 1.56 mm wall thickness, 7.5 m length) passed through the waveguides from the heart and lung machine in the control room into the magnet room, where the perfusate passed through a secondary heat exchanger (Plegiox, Getinge Rastatt, Germany) to balance for heat loss during transit. Effluent perfusate was pumped back into the reservoir.

**FIGURE 3:**
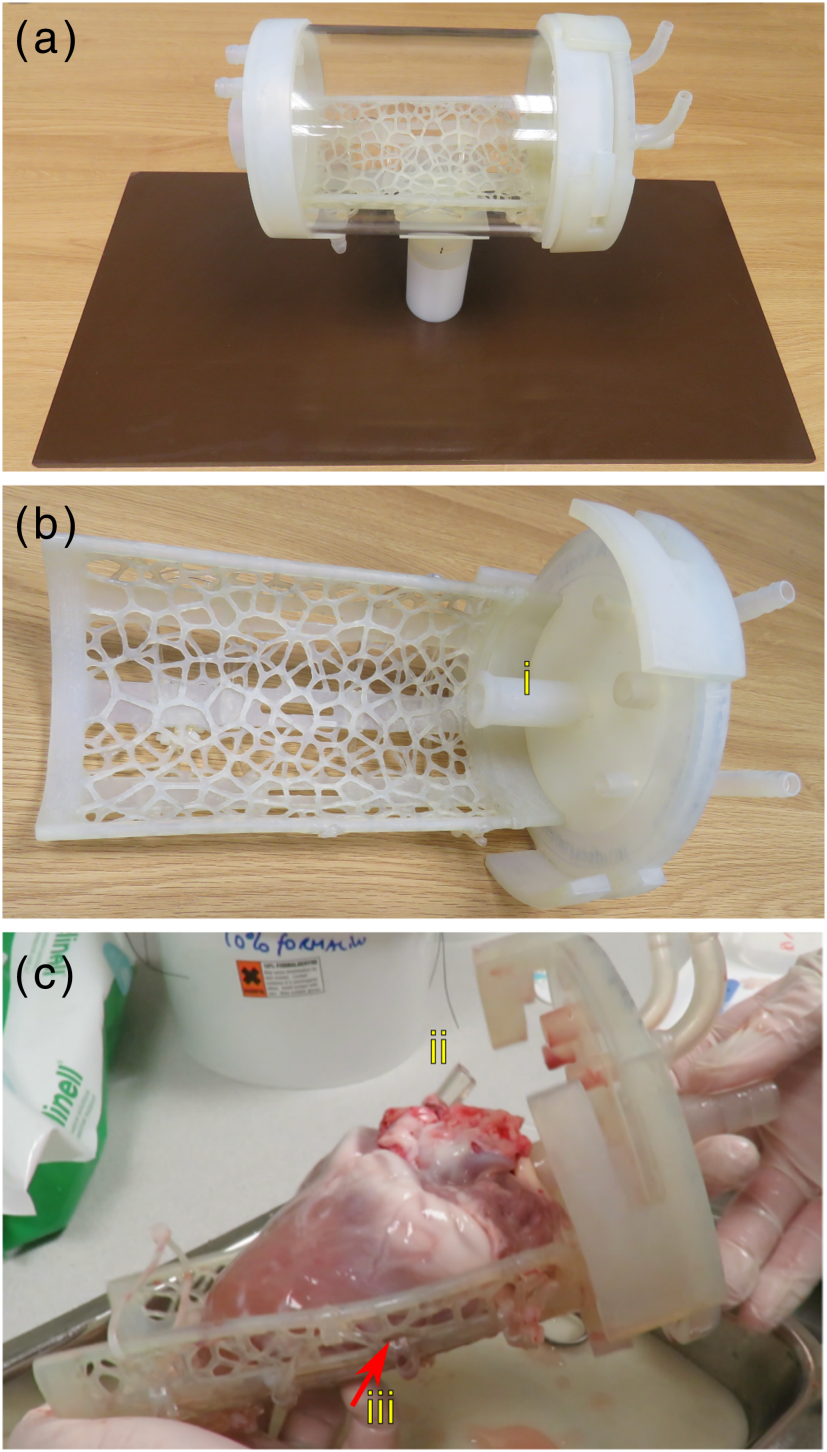
The custom 3D‐printed cardiovascular magnetic resonance (CMR)‐compatible perfusion chamber. This equipment was used for imaging the beating and arrested heart within the scanner (overview, (a)). The heart was supported at the isocentre of the scanner on a 3D‐printed mesh‐design tray (shown in (b)), which could be attached to the removable lid. The lid (b) included the aortic perfusion cannula (i). The left ventricle cavity vent is clearly visible in (c) (ii). Oil‐filled tubing (iii) on the bottom of the tray provided a fiducial reference for post‐CMR histology (c)

The heart was imaged within a custom 3D‐printed perfusion chamber (Figures 3 and S1). 3D‐printed components were manufactured using a stereolithograph system (Form 2, Formlabs Inc., Somerville, MA) from a hard‐wearing slightly flexible resin (Durable, Formlabs). Within the cylindrical chamber (130 mm diameter transparent acrylic tubing), the heart was suspended on a removable printed mesh supported at either end by the tubing end caps. One end cap was removable and twisted into place to seal the chamber. The seal allowed the heart to be imaged with the chamber filled with perfusate (aiming to reduce the susceptibility artefacts caused by the tissue–air interface) or empty. The aortic cannula was built into the removable end cap, which also featured ports for monitoring and stimulation cables. Effluent ports with barbed hose connectors were located on the base of the chamber. The chamber could be orientated with the axis aligned horizontally (parallel to the main scanner magnetic field, B0) or vertically, and sockets on the fixed end cap and middle of the tube connected to a base (PTFE cylindrical upstand and laminated plastic sheet [Tufnol] base) supporting the heart at approximately the magnet isocentre in either orientation. A single length of vegetable oil‐filled flexible silicone tubing (~2 mm internal diameter) was fixed to the underneath of the mesh heart support in a snake‐like pattern, crossing the midline of the printed mesh supporting the heart four times. This tubing provides four positions along the length of the heart (base‐apex) where there are fiducial markers that can be used to ensure that blocks cut for subsequent histology match the location of the imaging slice.

A small animal monitoring system (SA instruments, Stony Brook, NY) was used to monitor the heart surface temperature, perfusion pressure (in the aortic cannula) and ECG using needle electrodes in the myocardium. Pacing was provided by a standard clinical system (EV4543 miniature temporary cardiac pacemaker, Pace Medical, Devices Ltd, Hatfield, UK). An in‐house custom pulse lengthener was used to interface the pacing unit to an ECG waveform simulator (SA instruments), which was connected to the standard scanner ECG system, allowing triggering from the pacing signal as an alternative to the small animal ECG.

### 2.3 Langendorff perfusion

Upon arrival, hearts were removed from the packaging and placed into a 2‐L beaker containing ~1 L of fresh cardioplegia. To avoid air entering the coronary arteries, which could be difficult to exclude and result in subsequent coronary blockage, the heart was transferred rapidly and held with the aorta vertically upwards. The autologous blood was washed in a clinical autotransfusion cell saver system (Sorin Xtra, LivaNova, Mirandola, Italy) to provide red blood cells for increased oxygen‐carrying capacity in the perfusate later.

Video S2 shows video footage from one experiment. From the beaker of clean cardioplegia, the heart was rapidly transferred to a shallow plastic tray, where the cannula was inserted into the aorta, ensuring that the end of the cannula was not inserted beyond the coronary ostia. A cable tie was used to attach the heart to the cannula and a syringe containing fresh perfusate was used to fill the constantly draining aorta to avoid air bubbles entering the coronary arteries. To relieve any build‐up of fluid within the left ventricular cavity, a flexible tube was inserted into the left ventricle (LV) via a pulmonary vein and the left atrium crossing the mitral valve, and secured to the left atrium with a cable tie. Once the aortic cannula was fixed in place, the perfusion circuit (primed with room‐temperature perfusate) was quickly attached to the other side of the cannula and a final de‐airing was performed. The basic perfusate was a modified HEPES buffered Tyrode's solution, containing dextran to reduce the colloidal gradients. The constituents of all the perfusates used are described in Table S1.

Once the heart was satisfactorily connected to the perfusion circuit and the LV was vented, the perfusate temperature was increased to a temperature of 38°C at the heat exchanger/oxygenator and the flow rate was increased to achieve a target perfusion pressure of 50–80 mmHg (~250–350 ml/min flow). Initially, the effluent perfusate was discarded to wash out the cardioplegia. Fibrillating hearts were defibrillated with 5–20 J using a pair of internal paddles. Once the heart was beating without reverting to fibrillation, it was electrically paced at a minimum above its natural rate (i.e., at 80–100 beats per min); the perfusate was recirculated, and washed red blood cells were added to the perfusate. The estimated haematocrit was 25%–30%.

Next, the beating heart was inserted into the chamber and a flexible surface coil was attached to the surface of the chamber, bending to cover the cylinder. The chamber was then driven into the magnet isocentre using the scanner table.

### 2.4 CMR protocol

Imaging was performed using a 3‐T Siemens Skyra (Siemens Healthineers, Erlangen, Germany) with a four‐channel flexible matrix coil (flex large). Cardiac triggering was performed using the signal from the pacing unit. Cine imaging was performed using a retrospectively gated balanced steady state free precession (bSSFP) sequence to identify a midventricular short axis plane and the timings of the most contracted (peak systole) and the stationary period during the least contracted state (diastasis). Within the selected imaging plane, T1 mapping was performed using a standard precontrast modified Look Locker imaging (MOLLI) technique with a 5(3)3 protocol and bSSFP readout, and T2 mapping was performed using a T2‐prepared bSSFP technique with four T2 preparation times. DT‐CMR was performed in the same imaging plane using a STEAM echo planar imaging (EPI) sequence triggered to acquire the central k‐space data at either the most or least contracted states. Cine, T1 mapping, T2 mapping and DT‐CMR protocols are described in the supporting information.

Cine data were analysed using cvi42 (Circle Cardiovascular Imaging, Calgary). DT‐CMR data were processed using an in‐house tool to produce pixelwise diffusion tensors and the derived parameters^3^: mean diffusivity (MD), fractional anisotropy (FA), helix angle (HA) and absolute second eigenvector angle (E2A, a measure of sheetlet orientation). Numerical analysis of DT‐CMR parameters was performed in a region of interest covering the LV. b = 0 data were excluded from DT‐CMR analysis to minimise the confounding effects of perfusion.^21^ T1 and T2 maps were calculated using the standard product methods available on the scanner and a mean value was obtained from a midseptal region of interest.

### 2.5 Inducing arrest

Once acquisition of the DT‐CMR data was complete in the beating heart, the pacing was switched off and the perfusate was exchanged for an oxygenated blood‐free high potassium, calcium‐free modified Tyrode's solution (Table S1), to induce cardioplegia‐like arrest in a relaxed state. Initially this ‘slack’ perfusate was discarded to wash out the normal perfusate, but later the perfusate was recirculated. Arrest was confirmed by monitoring the heart using a real‐time cine acquisition. DT‐CMR and parametric mapping were repeated in the slack arrested heart. The perfusate was then switched a second time for another solution (Table S1) containing lithium rather than sodium to induce contracture in the arrested heart. Change in the myocardial shape was monitored via real‐time cine imaging and then DT‐CMR and parametric mapping image acquisitions were repeated using the same planes and protocols described above.

### 2.6 Histology

After CMR was complete the heart was removed from the perfusion chamber. Outside the magnet room, a transmural block of tissue (~2 x 3 cm rectangular cross‐section at the epicardium) was removed, using the location of oil‐filled tubes to match the location of the block to the DT‐CMR imaging slice. This block was fixed overnight in 5% formalin and then used in fluorescent confocal microscopy. The left ventricular cavity of the rest of the heart was filled with dental putty (hydrophilic vinyl polysiloxane light body, VPS HYDRO, Henry Schein, Gillingham, UK) through pulmonary veins via the left atrium and mitral valve to maintain its shape. The heart was then fixed in 10% neutral buffered formalin using two perpendicular lengths of surgical silk passed through the aortic vessel wall to suspend the heart within the container and avoid deformation where the heart sits on the base of the container.

The block of tissue removed for histology from one heart was cryosectioned at approximately 100‐μm thickness parallel to the epicardium to show the long axis of the cardiomyocytes in the imaging plane. Sections were stained with 4′,6‐diamidino‐2‐phenylindole (DAPI) (to stain nuclei) and a wheat germ agglutinin (WGA)‐conjugated fluorophore (to stain the cell membranes) within well plates to allow the stain to penetrate from both sides. 3D fluorescent confocal microscopy was performed using a Leica SP8 at 40x magnification with oil immersion. The orientation and dimensions of the cardiomyocytes were measured as described in the supporting information.

An additional neighbouring block of tissue was cut from the same heart after approximately 7 months in 10% neutral buffered formalin. This block was wax‐embedded and 313 contiguous 5‐μm sections were cut from the block in the radial–longitudinal plane to show the cardiomyocytes in cross‐section in the mesocardium and the sheetlet orientation. Masson trichrome staining was used to label the intracellular space red/pink, collagen blue and nuclei black. Slides were imaged at 20x magnification on a bright field automated slide scanner (Nanozoomer S210, Hamamatsu, Hamamatsu City, Japan). Images were coregistered and structure tensor analysis was used to extract the sheetlet orientation (see the supporting information for details).

## 3 RESULTS

Of the total of 10 hearts that were harvested and perfused, six achieved a consistent beating state (successful hearts), including the last five hearts. The median transport time was 3 h 10 min (range 2–4 h) and the median ischaemic time was 3 h 50 min (3 h 15 min–5 h 40 min). A temperature of 38°C was measured on the surface of the perfused heart. Table S2 summarises the probable reasons for success or failure in each heart. Imaging of the beating heart including DT‐CMR was performed in hearts #7–#10, imaging for hearts #9 and #10 was performed within the chamber and with the full protocol (beating, slack and then contracted arrest), while histology was performed in heart #10. The median ischaemic time was 4 h 30 min for the unsuccessful hearts (3 h 30 min–5 h 40 min) and 3 h 55 min for the successful hearts (3 h 15 min–5 h 25 min), which was not significant (Mann–Whitney rank‐sum, *p* = .35). In the successful hearts, the median time from the first contraction to the end of pacing was 1 h 35 min (1 h 17 min–2 h 19 min), and the experiment was stopped in every successful case while the heart was still beating.

Experience from the initial experiments (hearts #1–#5) led to protocol developments and an increasingly successful experiment. The initial experimental setup had the hearts hanging vertically from the aorta (N = 2) and the LV cavity was unvented. Autologous blood was not added to the perfusate until the sixth heart and we initiated a careful connection procedure to avoid air entering the coronary arteries from heart #6 (as described in the Methods). The ability to trigger from the pacing signal or ECG was found to be useful, due to difficulties in obtaining reliable ECG signals in the scanner (later found to be the result of an intermittently faulty ECG power cable). Gradient interference with the triggering signal led to difficulties in detecting the R‐wave during the diffusion time in the STEAM sequence for three of the four hearts DT‐CMR was performed in. As an alternative, a single R‐wave was used to trigger the sequence and the measured pacing interval was set as a fixed time between the two diffusion‐encoding gradients.

Hearts were successfully arrested by ceasing to pace and switching the perfusate to the slack formulation.

**FIGURE 4:**
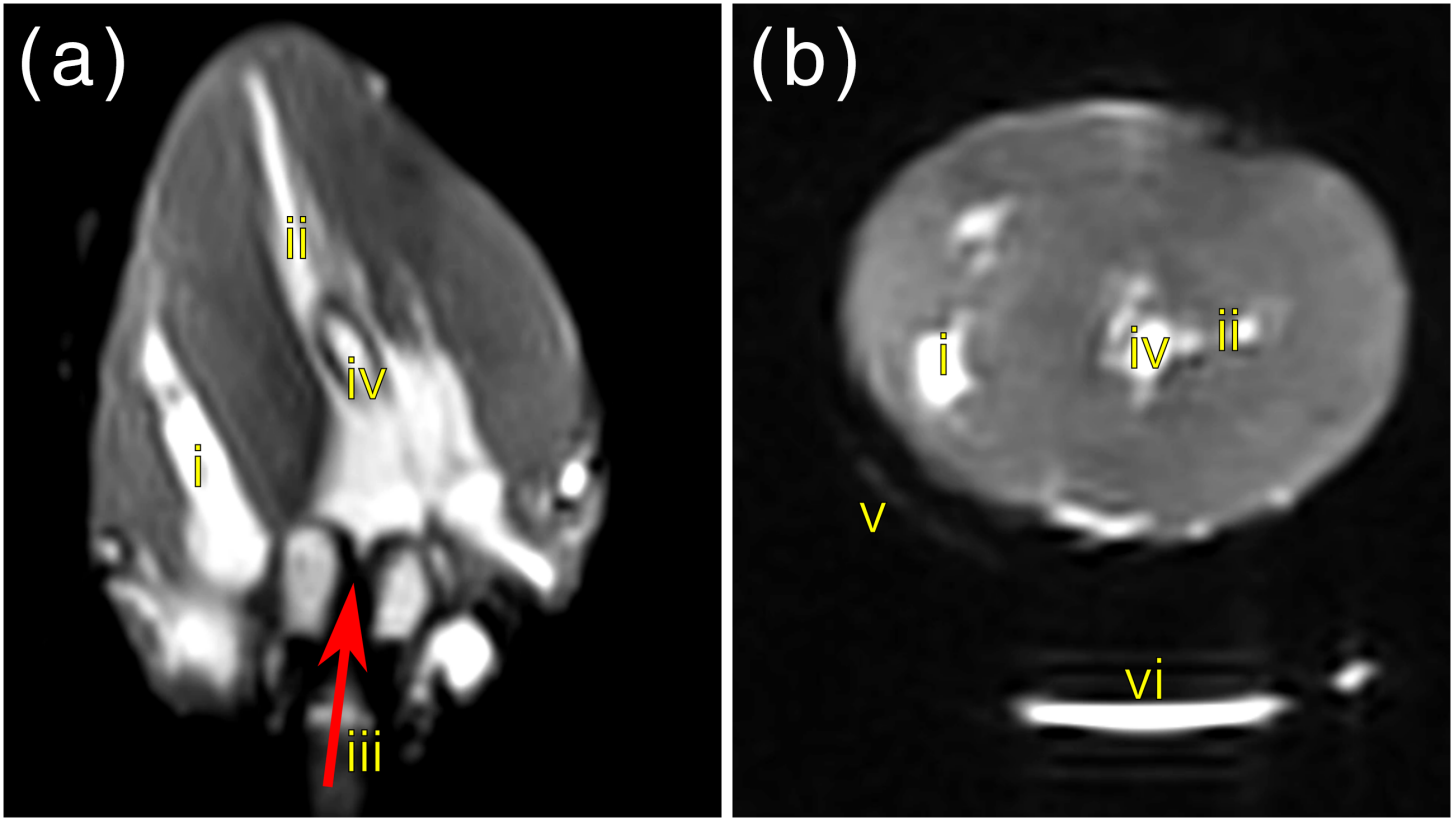
Still frames from cine cardiovascular magnetic resonance (CMR) in the beating Langendorff‐perfused heart. Long (a) and short axis (b) still frames from cine acquisitions in heart #10. Both images clearly show the right (i) and left (ii) ventricular blood pools. The long axis, approximately equivalent to a four‐chamber view (a), shows the perfusion through the aorta ((iii), the red arrow indicates the perfusate flow direction) and the location of the left ventricle cavity vent (iv). The short axis image (b) also shows effluent perfusate in the bottom of the chamber (vi) before it is drained with the secondary pump circuit and the oil‐filled tubing (v). Video S3 shows the equivalent beating heart images

**FIGURE 5:**
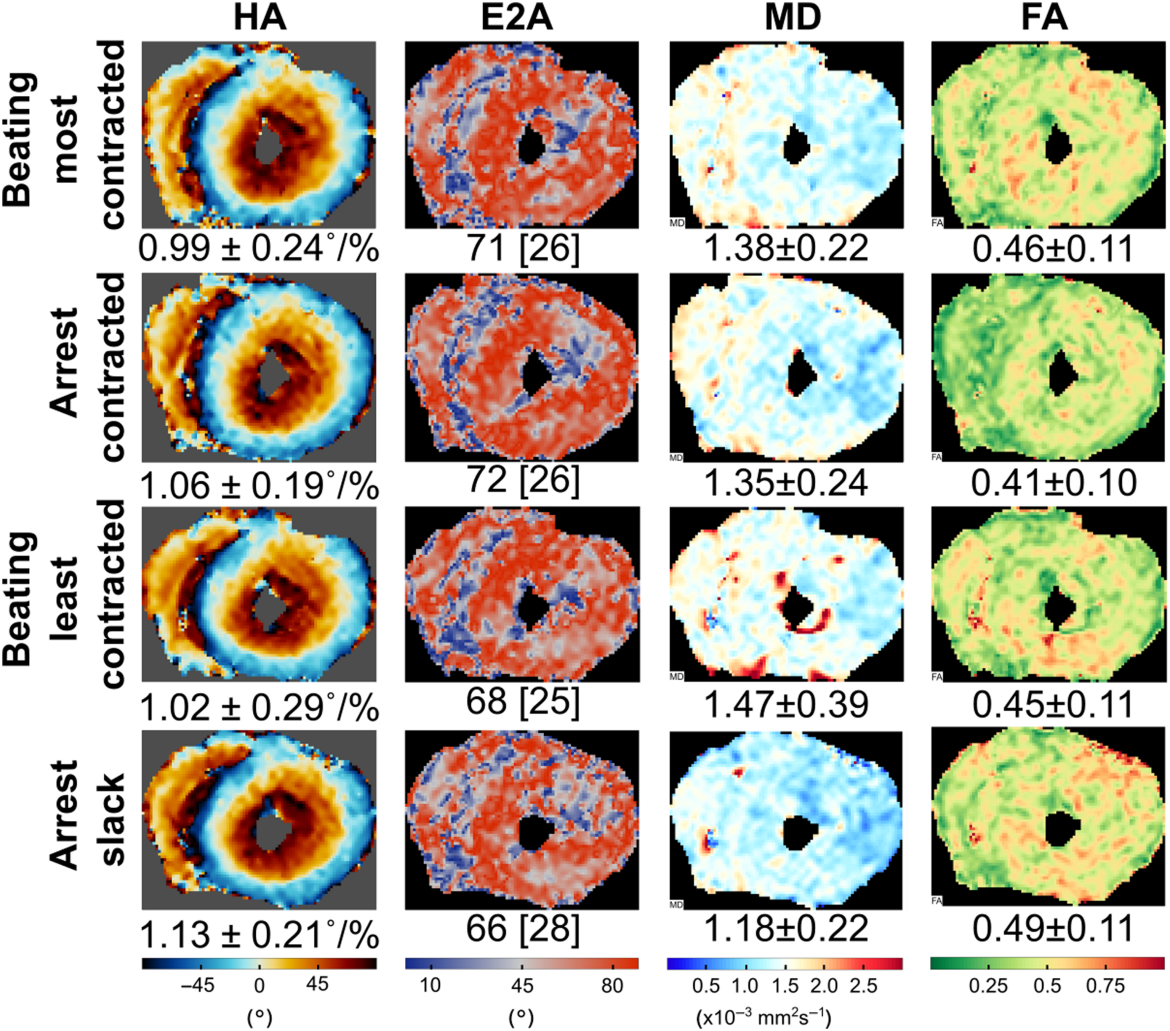
Diffusion tensor cardiovascular magnetic resonance (DT‐CMR) results from heart #10 acquired in the most and least contracted cardiac phases in the beating heart and in both slack and contracted arrested states. There is good agreement between the equivalent arrested and beating states, but little difference between the two contractile states (most contracted beating vs. least contracted beating) and (most contracted arrest vs. least contracted arrest) in both cases. The mean (median for E2A) and standard deviation (interquartile range for E2A) over the left ventricular myocardium is shown below each map, except for the helix angle (HA), where the mean transmural helix angle gradient is shown

**FIGURE 6:**
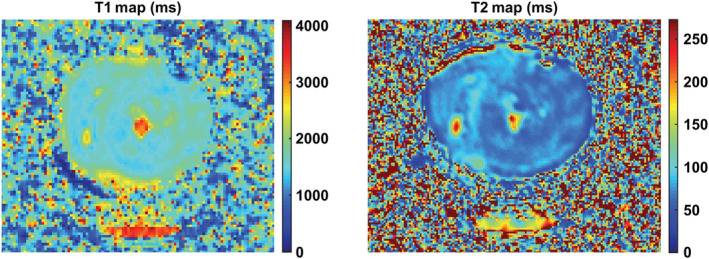
Example T1 and T2 maps from heart #10. T1 data were acquired using the Modified Look Locker Imaging method and T2 data using a T2 preparation‐based method. Data were acquired ~1 h after the heart began contracting after reperfusion

**FIGURE 7:**
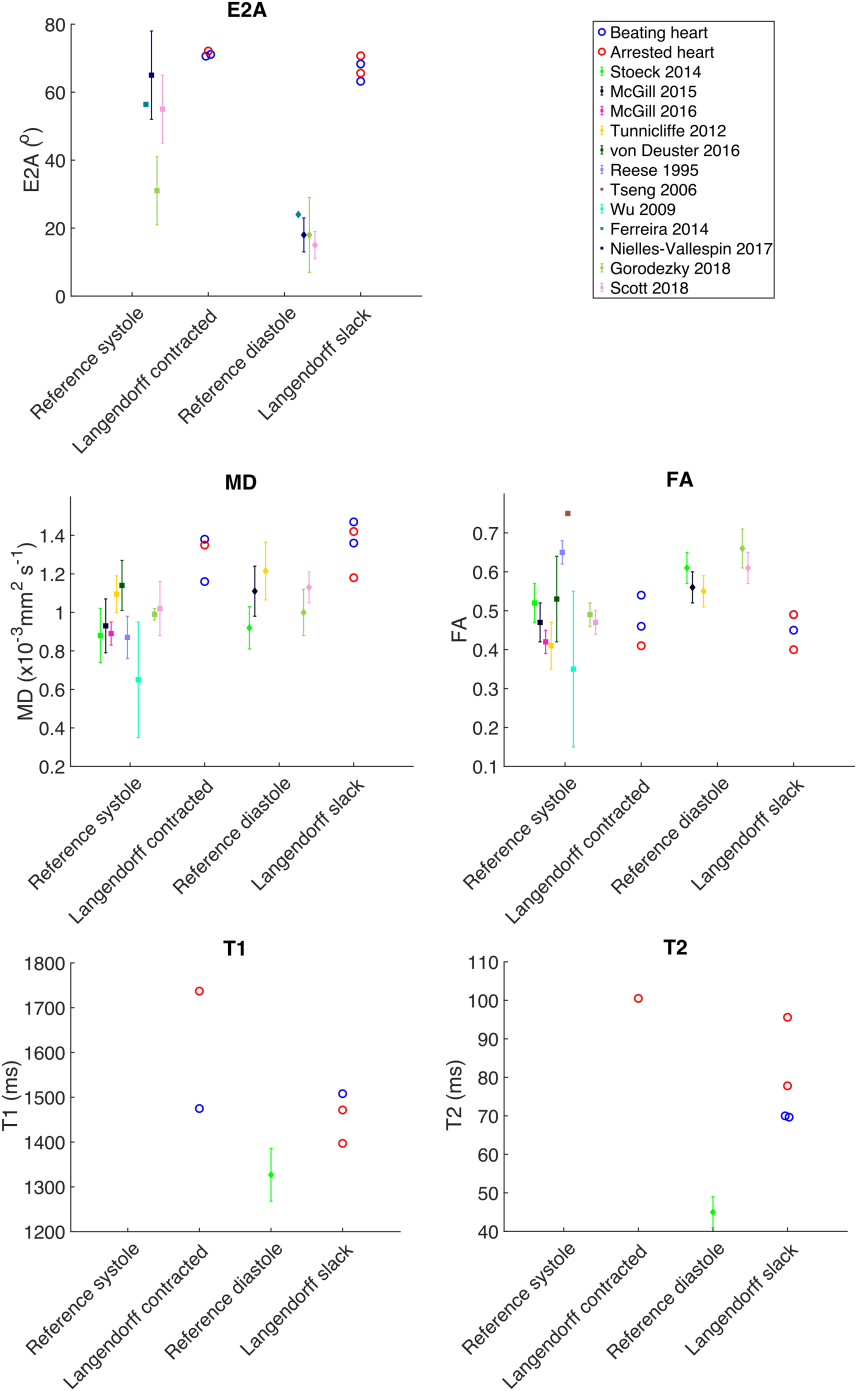
A comparison of T1, T2 and parametric mapping between the beating and arrested hearts with comparison with literature values. Reference T1 and T2 values were obtained in the study described in Scott et al.^22^ Langendorff values were obtained in hearts #9 (most contracted, least contracted and arrested slack) and #10 (beating most contracted and least contracted and arrested contracture). T2 mapping beating heart data were acquired in the most contracted phase, and T1 mapping data were acquired in least contracted for heart #9 and most contracted for heart #10, because of triggering problems in the least contracted phase. Literature values are plotted as mean ± standard deviation or median ± interquartile range over the cohort as available,^2^, ^3^, ^4^, ^9^, ^22^, ^23^, ^24^, ^25^, ^26^, ^27^, ^28^, ^29^ whereas the Langendorff values are plotted as individual points for the mean left ventricular value (median for E2A) in each pig heart. Langendorff beating heart values are shown as contracted (referred to as ‘most contracted’ in the text) or slack (referred to as ‘least contracted’ in the text)

**FIGURE 8:**
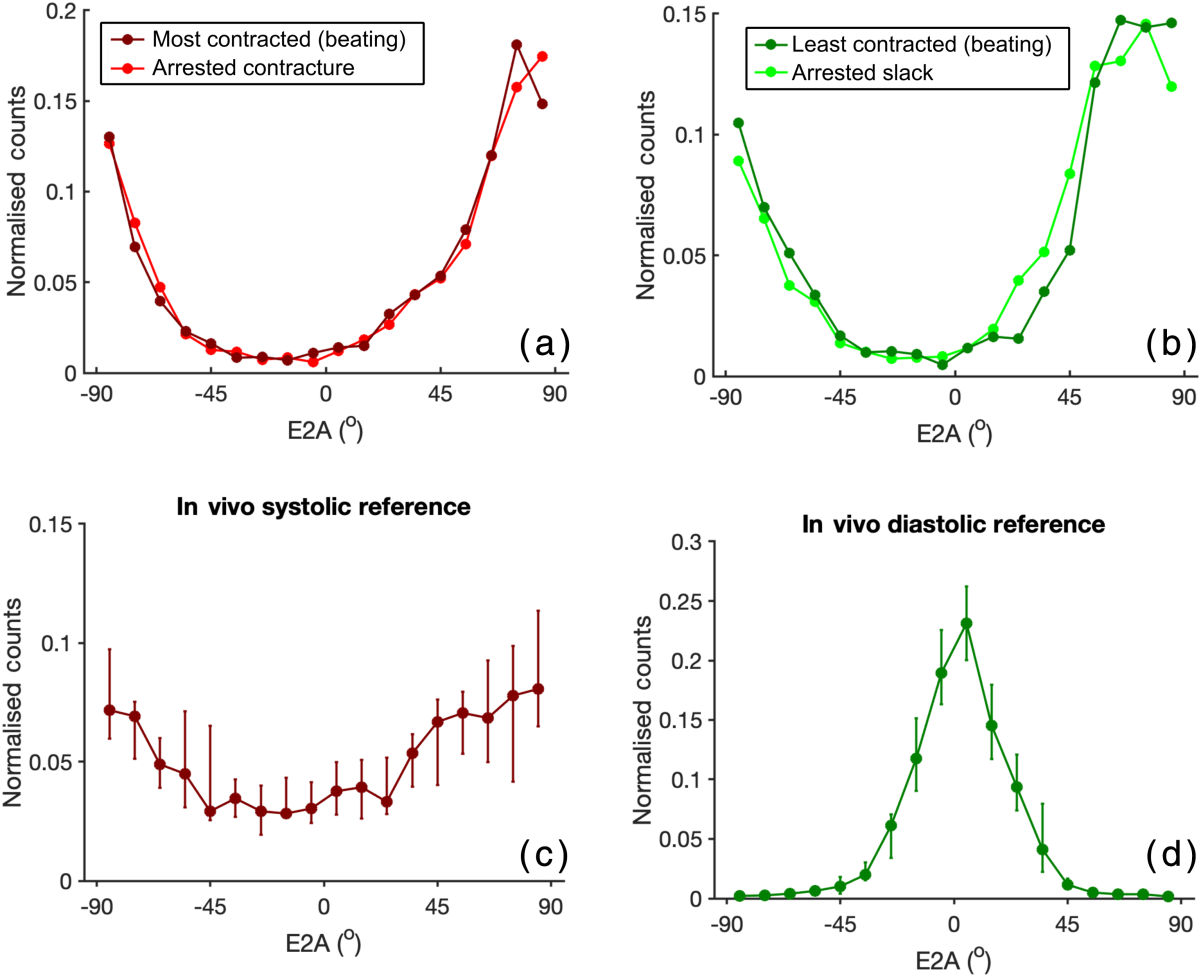
E2A distribution within the left ventricular myocardium in corresponding beating and arrested hearts ((a) contracture arrest/beating most contracted and (b) slack arrest/beating least contracted) with comparison to equivalent data acquired in vivo in a set of different porcine hearts (N = 6 systole (c), N = 5 diastole (d)). The Langendorff‐perfused heart does not achieve a diastolic‐like E2A distribution in the least contracted state. Reference data (c and d) replotted as median ± interquartile ranges are from Ferreira et al.^12^

Figure 4 shows still frames from bSSFP cine acquisitions in heart #10 and the corresponding videos are provided in Videos S3 and S4. The image quality was good and an approximate LV ejection fraction of 10% was calculated from the short and long axis cine data using the Simpson method. LV wall thickness analysis (10 slices, 50 chords per slice excluding papillary muscles) provided values of (mean ± standard deviation) 17.2 ± 4.2 mm in diastole and 17.1 ± 4.2 mm in systole, with peak global strains (2D feature tracking analysis) of 0.2%, −0.2% and −1.8% in the radial, circumferential and longitudinal orientation, respectively. Figure 5 shows DT‐CMR results from heart #10 both in the most and least contracted cardiac phases and contracted and slack arrested states. While the DT‐CMR parameter maps appear visually similar, and the DT‐CMR parameters averaged over the LV (Figure 5) are similar in the corresponding beating and arrested states, there was little difference between the most contracted beating phase and the contracted arrest, and the least contracted beating phase and the slack arrest. Figure 6 shows example T1 and T2 maps from heart #10 acquired while beating, and Figure 7 shows a comparison of average midseptal T1 and T2 and slice‐averaged DT‐CMR parameters in the beating and arrested hearts (#9 and #10) with literature values from similar techniques in healthy humans. HA was quantified as the helix angle gradient in degrees per percentage wall thickness, with mean values ranging from 0.99 ± 0.24°/% to 1.13°/%. MD was elevated (1.16–1.47 x 10^−3^ vs. 0.65–1.22 x 10^−3^ mm^2^ s^−1^ comparable average literature values^2^, ^22^, ^23^, ^24^, ^25^, ^26^, ^27^) and FA was reduced (range 0.40–0.54 vs. 0.35–0.75 comparable literature values^2^, ^22^, ^23^, ^24^, ^25^, ^26^, ^27^) in the Langendorff‐perfused hearts. Both T1 (1397–1737 ms) and T2 (70–101 ms) were higher than reference values from healthy humans acquired on the same scanner using the same sequence (T1 = 1327 ± 59 ms, T2 = 45 ± 4 ms^22^), and median LV E2A was systolic‐ or hypersystolic‐like in most contracted (70.6–72.1 vs. 31–65° from comparable previous studies^3^, ^22^, ^23^, ^24^, ^27^) and least contracted Langendorff hearts (63.2–70.7 vs. 15–24° from comparable previous studies). Analysis of the distribution of E2A in the short axis slice imaged shows a good correspondence between the equivalent beating and arrested data (Figure 8). However, the distributions from both contracted and slack hearts demonstrate the U‐shaped histograms also obtained from literature data^12^ in a separate set of porcine hearts imaged in vivo in systole (Figure 8c), and which are typically found in systolic data from healthy in vivo human hearts.^3^

Heart #9 was weighed on both arrival and after completion of CMR, before removing blocks for histology and fixation. This heart increased in mass from 315 to 470 g.

**FIGURE 9:**
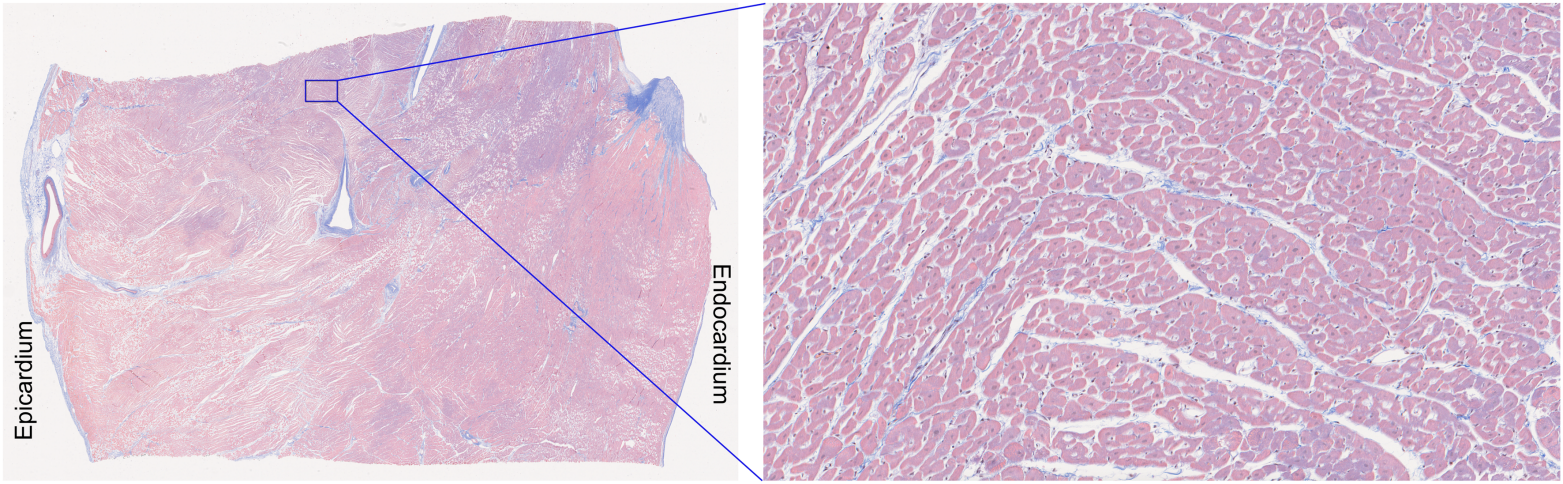
Example bright field histology. Masson‐stained 5‐μm sections in the longitudinal–transmural plane with a magnified region (right). Cytoplasm stains red/pink, collagen blue and nuclei black. The full stack of images is shown in Video S5

**FIGURE 10:**
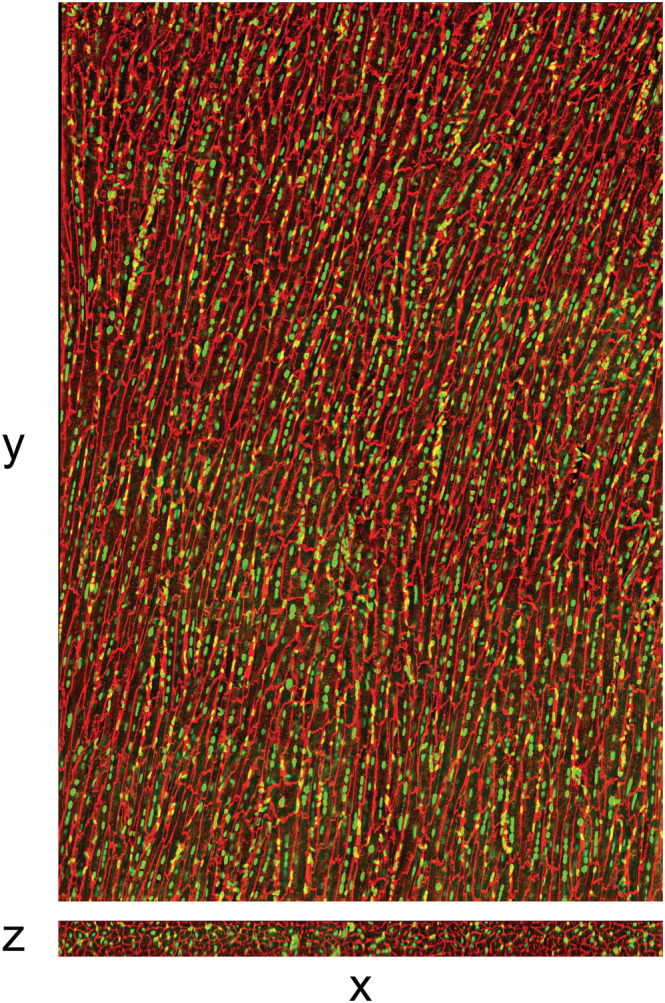
Example fluorescent confocal histology images. Images are from 3D fluorescent confocal acquisition of a thick section ~100‐μm stained with wheat germ agglutinin (red, cell membranes) and DAPI (green, nuclei). Sections were cut parallel to the epicardial surface. The 3D acquisition allows resectioning in any plane. The images shown were acquired using a tile scan (four columns, six rows) method and the in‐plane field of view is ~1 x 1.5 mm^2^ after combination (as shown). A movie of the same data is shown in Video S6

Figure 9 shows a typical Masson‐trichrome stained 5‐μm section cut transmurally from heart #10 in a region determined to intersect with the DT‐CMR imaging slice using the oil‐filled tubing. The magnified section from the mesocardium shows the cardiomyocytes cut in cross‐section and their arrangement into sheetlets, separated by collagen‐lined shear layers. The full stack of 313 images is shown in Video S5. The sheetlet angle distribution obtained using the structure tensor analysis is shown in Figure S5 and the median absolute sheetlet angle from all slices is 59.9° (see the supporting information for more details). Figure 10 shows a demonstration of the 3D confocal fluorescence data that were acquired for a section of tissue from a neighbouring block in the same heart, and the stack of slices is shown as a video in Video S6. The median length, mean width and mean height of the cardiomyocytes (as defined in the supporting information) were 156.5, 24.5 and 17.4 μm, respectively.

## 4 DISCUSSION

We have developed a CMR model for direct comparison of beating and arrested whole large hearts in a similar state of contraction. In the future this model will provide new opportunities to study the effects of cardiac contraction and strain on the myocardium and the CMR methods used to investigate this. Uniquely, DT‐CMR provides insights into cardiac microstructure and its function and this novel preparation provides the ability to monitor the evolution of DT‐CMR during cellular contraction with data from the same hearts, both beating and arrested, supported by subsequent colocalised histology.

The use of a heart transplant‐like protocol for the harvesting, protection and transport of the hearts allows the preclinical and imaging facilities to be located in different institutions in different parts of the country, making synergistic use of the equipment and expertise in both locations. We were able to establish a stable beating heart in six of the 10 hearts harvested. While the overall 60% success rate appears low, it represents the necessary learning curve in the initial stages of the study. We achieved 100% success in establishing the final five hearts due to protocol refinements and the final two test hearts were successfully imaged using all methods in the perfusion chamber. Although this is a single‐site experience, the success rate highlights the number of animals that should be planned for when developing such a model.

Hearts were imaged in a beating state and then arrested in a slack state using a cardioplegia‐like perfusate followed by a state of contracture using a lithium‐based perfusate. In future work, the use of excitation‐contraction uncouplers, including 2,3‐Butanedione monoxime, could be investigated as a method to avoid motion artefacts without a full cardiac arrest.^30^

DT‐CMR results, including HA, FA, MD and E2A, appear similar between the equivalent arrested and beating hearts. There was, however, little difference between the most contracted and least contracted states, either in the beating or arrested hearts. The cine data suggest that the heart maintains a contracted or hypercontracted state throughout the cardiac cycle, with relatively poor function, which is consistent with the large E2A angles observed in both the most and least contracted states and our previous findings of high E2A in systole.^4^ We have referred to the two cardiac phases at which DT‐CMR was performed as ‘most contracted’ and ‘least contracted’, as we do not believe that these two states truly reflect systole and diastole, respectively. The elevated MD and low FA in the least contracted state are all consistent with oedema and the 50% increase in mass of heart #9 would be in keeping with this. While inversion and T2 preparation times were not optimised for the long T1 and T2 values of the Langendorff hearts in this work, the fact that the values from these protocols are elevated is still consistent with increased myocardial water content. Oedema is a recognised limitation of Langendorff perfusion models, particularly with crystalloid perfusates. Future work will look to address this issue and achieve in vivo‐like bulk cardiac function by harvesting larger volumes of blood from the animals to provide in vivo‐like haematocrit values and by loading the ventricle using a balloon, as in other studies,^31^, ^32^ or modifying the perfusion circuit to operate in working heart mode, providing preload and afterload. It is hoped that these additional developments will facilitate the quantification of the effects of in vivo levels of cardiac strain on STEAM DT‐CMR data via isolated perfused hearts.

In addition to providing the first demonstration of DT‐CMR performed in an ex vivo beating heart, as far as we are aware this is the first study to also show cine CMR data from an isolated perfused beating pig heart and the first to use fiducial markers to facilitate colocalised histology. We developed a unique MRI‐compatible Langendorff perfusion chamber using 3D printing technology, which supports the heart and separates the heart from the receive coil, while providing a high receive coil fill factor and providing fiducial markers for the subsequent histology. The chamber can be filled with effluent perfusate to reduce susceptibility differences and is designed to allow access for monitoring and pacing systems. Here, we chose to image the heart surrounded by air to avoid the potential effects of the external fluid pressure and fluid inertia on myocardial motion. No evidence of substantial off‐resonance artefacts was present in the DT‐CMR data. Although not shown in this work, the chamber axis could also be rotated through 90° to study the effects of magnetic field orientation on the CMR data.

The results of the analysis of the histology data will provide important input into ongoing computational simulations of DT‐CMR.^33^ Studies in rats have measured average cardiomyocyte dimensions of 140.7 ± 8.5 μm (length), 30.3 ± 1.3 μm (width) and 12.2 ± 1.0 μm (height) with an aspect ratio of 2.5 ± 0.2,^34^ with similar results obtained in swine.^35^ These results are consistent with the measurements obtained here of 156.5, 24.5 and 17.5 μm (length, width and height, respectively), although our results suggest a more circular cross‐section (aspect ratio 1.49 ± 0.25 here). The histological measurement of the sheetlet angle (median absolute angle 59.9°) was consistent with a contracted myocardium and only slightly lower than the median LV E2A measured in the arrested contracted state at 72 (26)°.

Previous studies have developed MRI‐compatible Langendorff‐perfused porcine heart models to study the effects of ischaemia, preservation and reperfusion protocols,^32^, ^36^, ^37^, ^38^, ^39^, ^40^, ^41^, ^42^ but imaging was performed with either arrested hearts or was ungated. Schuster et al.^19^, ^31^, ^43^ developed an MRI‐compatible beating heart Langendorff system for the validation of CMR‐based quantitative perfusion. ECG‐triggered images were acquired in beating hearts, and selective perfusion of the left and right coronary artery systems with a dialysis filter in the perfusion circuit facilitated multiple first pass perfusion experiments in the same heart. More recently, an MRI‐compatible system allowing gated imaging of beating large animal hearts (pig and sheep) in both Langendorff perfusion and working heart modes was presented.^44^ Working isolated heart models load the heart, providing both preload and afterload, thereby producing a more physiologically realistic heart at the expense of a more complex experimental setup. However, none of these models assessed myocardial microstructure or used the isolated perfused heart to assess the effects of motion or strain on the CMR measures. Hales et al.^20^ used DT‐CMR of Langendorff‐perfused rat hearts to identify changes in sheetlet (via the third eigenvector orientation) and cardiomyocyte orientation between a slack arrest (high potassium) and a contracted arrest (lithium chloride). Lohezic et al.^45^, ^46^ developed this model further to allow assessment in multiple arrested states, but neither study performed DT‐CMR in a beating heart.

Previous studies have often used a heated perfusion chamber,^19^ but we instead used an additional heat exchanger to compensate for heat loss along the long perfusion tubing lines, which allows the CMR receiver coil to wrap closely around the heart and provide more signal. The use of electrical equipment in the magnet room often produces image artefacts, and one setup described in the literature used long drive rods between the pump motors and pump heads travelling through the waveguide to keep the pump motors out of the Faraday cage,^31^ but we chose to pass the perfusion tubing through the waveguide and rewarm the perfusate instead, which avoids the cost and inconvenience of the split motor–pump head setup.

In prior work, we compared DT‐CMR in beating pig hearts with the same hearts arrested in situ, followed by ex vivo DT‐CMR and histology for validation of DT‐CMR–derived measures of sheetlet orientation.^4^ However, each heart was arrested in either a slack (potassium chloride) or contracted (lithium chloride) state, while each Langendorff‐perfused heart could be imaged in various stages of contraction and the model could be modified in future to load the arrested heart.^45^ Further advantages of the isolated perfused heart setup over in vivo and in situ imaging include the high receive coil fill factors, which result in high SNR and the avoidance of breath‐holding or respiratory motion. As we have demonstrated, the use of an isolated perfused heart allows straightforward colocalisation of the imaging data and blocks of tissue used for histology. Future studies may include investigation of the effectiveness of motion compensation provided by the STEAM sequence by comparing the phase images between equivalent beating and arrested heart data, in a manner similar to Stoeck et al.^47^

Beyond the investigation of CMR methods, we believe this CMR‐compatible isolated perfused beating large animal heart model has potential use in assessing myocardial tissue engineering methods, cardiac regeneration therapy and in joint imaging–electrophysiology studies.^48^

There are a number of limitations to this study, in addition to the myocardial oedema and apparent failure to achieve microstructural relaxation, as discussed above. Eight out of 10 hearts were used in refining the methods and a full protocol was only performed in one heart, so a full statistical analysis would therefore be of little value. Increasing the number of hearts is difficult within the constraints of the replacement, reduction and refinement. Others have sourced hearts for isolated perfusion from abattoirs as meat by‐products,^49^ although this does reduce control over the harvesting process and requires a cooperative partner abattoir. We have not investigated the effect of the order of arrest, although the cardioplegia‐like nature of the ‘slack’ perfusate suggests that this should logically come before the lithium‐induced ‘contracture’ arrest. Future work should investigate the effects of the period of the slack arrest on the contracted arrest.

While we used the oil‐filled tubing as a fiducial marker for colocalisation of the histology and imaging slices, the tubing is slightly displaced from the epicardium. The precision of colocalisation should be quantified in follow‐up studies and the precision may be improved by using additional markers on the opposite side of the heart. Basic 2D feature tracking was used to provide estimates of global strain in the beating hearts. Initial tests using our preferred strain measurement sequence, spiral cine DENSE,^50^ demonstrated severe artefacts due to the combination of the spiral readout and the change in magnetic susceptibility between the heart and the surrounding air. Future work will look to optimise DENSE sequences or evaluate tagging sequences to provide regional strain measures in isolated perfused hearts.

In conclusion, we have described the development of a large animal perfused beating heart preparation for analysis of the effects of motion on CMR data, including microstructural measures obtained from DT‐CMR. The use of isolated perfused hearts allows colocalisation of CMR and histology data, and separation of the CMR and preclinical harvesting facilities by several hours of travelling time.

## Supporting information


**Table S1:** Perfusate constituents.
**Table S2:** A summary of the success of each of the 10 experiments.
**Figure S1:** Images from the computer aided design files for the MRI compatible perfusion chamber shown in Figure 3. The 3D printed parts are colour coded in A and B. All parts are 3D printed apart from the seals shown in black in A and the transparent acrylic tube shown in A and B. The lid of the chamber is shown in purple, with the barbed connection to inflowing perfusate circuit shown in turquoise and the additional barbed connections for monitoring systems in white. The cannula inserted into the aorta extends out from the turquoise connector into the main chamber (not shown). The structural elements of the support bed are shown in green (A and B) and the same bed is shown with the Voronoi design covering the top and bottom of the structure shown in green in C. The 3D printed lid shown in purple connects to the flange shown in yellow, twisting into place to seal via the rubber o‐rings shown in black. The 3D‐printed chamber support post is shown in red and connects to a machined PTFE post on the flat base holding the chamber. The bottom of the chamber is shown in blue and the barbed effluent ports are visible on the bottom of the chamber. The corresponding STL files used for 3D printing are available on request.
**Figure S2:** Examples of the manual measurements made on the confocal data set. In the original imaging plane (A), the cardiomyocyte length (l) and diameter (d) was measured with the in‐plane rotation (a) of the cardiomyocytes relative to the vertical axis of the image. In the first resliced orientation (B) the rotation of the cardiomyocytes through the image plane was measured (e). In the second resliced orientation (C), the width (w), parallel to the original imaging slice orientation and height (h) perpendicular to the original imaging plane were measured.
**Figure S3:** Results of the measurements performed on the confocal data in the original imaging plane (A‐C) and in planes created by reslicing the data in perpendicular orientations (D and E). The angle in A is defined in supplementary Figure 1.A as “a”. The length shown in B and the diameter shown in C and defined in supplementary Figure 1.A as “l” and “d” respectively. The elevation angle shown D is defined in supplementary Figure 1.B as “e” and the height and width shown in E are defined in supplementary Figure 1.C as “w” and “h” respectively.
**Figure S4:** Structure tensor analysis applied to an example slice. The images are converted to grayscale (A) and a region is selected that shows the cardiomyocytes in cross section in all slices (red region in A). The selected region is shown in B with the sheetlet orientation extracted using the structure tensor analysis superimposed in magenta. A zoomed in region of the image in B (highlighted in green) in shown in C. The magenta lines are seen to align with the sheetlet orientation.
**Figure S5:** The results of the structure tensor analysis applied to the brightfield microscopy data presented as a histogram. The histograms from the individual slices are shown in blue and the mean over all slices is shown in red. The sheetlet orientation plotted here is defined as the angle from the vertical orientation in the images, with a clockwise angle shown as positive. Assuming that the local wall tangent is vertical in the images, this definition matches that of the E2A used in DTCMR.

**Video S1:** The procedure used to arrest the hearts. After sternotomy and the heart is exposed, the superior and inferior vena cava are clamped and the heart is drained of blood. The aorta is then clamped above the coronary sinus and cardioplegia is administered into the aortic root, forcing it down the coronary arteries and inducing ventricular fibrillation followed by arrest. After the video stops, the heart is rapidly excised and stored in a container of cold cardioplegia surrounded by ice.

**Video S2:** A summary of the procedure used to initiate perfusion, establish stable contraction and get the beating heart into the MRI scanner. Filming performed for heart 10.

**Video S3:** bSSFP cine images acquired in a long axis, 4 chamber‐like view (left) and a mid‐ventricular short axis (right) from heart #10. Still labelled images are shown in Figure 3.

**Video S4:** A stack of bSSFP images extracted from cine acquisitions in heart #10. Images were all acquired immediately after triggering from the pacing signal with an 8 mm slice thickness and 2 mm gap between adjacent slice edges.

**Video S5:** A stack of 313 contiguous 5 μm Masson stained sections cut from a transmural block (radial – longitudinal plane) co‐localised with the imaging plane. Images are registered as described in the supplementary material.

**Video S6:** A stack of 3D confocal fluorescent images in a plane parallel to the epicardium. The 100 μm section was stained with DAPI (green) for nuclei and WGA (red) for the cell membranes. A 6x4 tilescan was used to obtain the images shown.

## References

[1] Scott AD , Keegan J , Firmin DN . Motion in cardiovascular MR imaging. Radiology. 2009;250(2):331‐351. doi:10.1148/radiol.2502071998 19188310

[2] Stoeck CT , Kalinowska A , von Deuster C , et al. Dual‐phase cardiac diffusion tensor imaging with strain correction. PLoS ONE. 2014;9(9):e107159. doi:10.1371/journal.pone.0107159 25191900 PMC4156436

[3] Ferreira PF , Kilner PJ , McGill LA , et al. In vivo cardiovascular magnetic resonance diffusion tensor imaging shows evidence of abnormal myocardial laminar orientations and mobility in hypertrophic cardiomyopathy. J Cardiovasc Magn Reson. 2014;16:87. doi:10.1186/s12968-014-0087-8 25388867 PMC4229618

[4] Nielles‐Vallespin S , Khalique Z , Ferreira PF , et al. Assessment of myocardial microstructural dynamics by in vivo diffusion tensor cardiac magnetic resonance. J Am Coll Cardiol. 2017;69(6):661‐676. doi:10.1016/j.jacc.2016.11.051 28183509 PMC8672367

[5] Khalique Z , Ferreira PF , Scott AD , et al. Deranged myocyte microstructure in situs inversus totalis demonstrated by diffusion tensor cardiac magnetic resonance. JACC Cardiovasc Imaging. 2018;11(9):1360‐1362. doi:10.1016/j.jcmg.2017.11.014 29361490

[6] von Deuster C , Sammut E , Asner L , et al. Studying dynamic myofiber aggregate reorientation in dilated cardiomyopathy using in vivo magnetic resonance diffusion tensor imaging. Circ Cardiovasc Imaging. 2016;9(10):e005018. doi:10.1161/CIRCIMAGING.116.005018 27729361 PMC5068188

[7] Gotschy A , von Deuster C , van Gorkum RJH , et al. Characterizing cardiac involvement in amyloidosis using cardiovascular magnetic resonance diffusion tensor imaging. J Cardiovasc Magn Reson. 2019;21(1):56. doi:10.1186/s12968-019-0563-2 31484544 PMC6727537

[8] Edelman RR , Gaa J , Wedeen VJ , et al. In vivo measurement of water diffusion in the human heart. Magn Reson Med. 1994;32(3):423‐428. 7984077 10.1002/mrm.1910320320

[9] Reese TG , Weisskoff RM , Smith RN , Rosen BR , Dinsmore RE , Wedeen VJ . Imaging myocardial fiber architecture in vivo with magnetic resonance. Magn Reson Med. 1995;34(6):786‐791. 8598805 10.1002/mrm.1910340603

[10] Nielles‐Vallespin S , Ferreira PF , Gatehouse PD , et al. Time‐resolved in vivo cardiac diffusion tensor MRI of the human heart. Proc Int Soc Magn Reson Med Annu Meet Salt Lake City, Utah, USA. 2013:0479.

[11] Reese TG , Wedeen VJ , Weisskoff RM . Measuring diffusion in the presence of material strain. J Magn Reson B. 1996;112(3):253‐258. 8812913 10.1006/jmrb.1996.0139

[12] Ferreira PF , Nielles‐Vallespin S , Scott AD , et al. Evaluation of the impact of strain correction on the orientation of cardiac diffusion tensors with in vivo and ex vivo porcine hearts. Magn Reson Med. 2018;79(4):2205‐2215. doi:10.1002/mrm.26850 28734017 PMC5776058

[13] Stoeck CT , von Deuster C , Genet M , Atkinson D , Kozerke S . Second‐order motion‐compensated spin echo diffusion tensor imaging of the human heart. Magn Reson Med. 2015;75(4):1669‐1676. 26033456 10.1002/mrm.25784

[14] Gamper U , Boesiger P , Kozerke S . Diffusion imaging of the in vivo heart using spin echoes‐considerations on bulk motion sensitivity. Magn Reson Med. 2007;57(2):331‐337. doi:10.1002/mrm.21127 17260376

[15] Tseng WY , Reese TG , Weisskoff RM , Wedeen VJ . Cardiac diffusion tensor MRI in vivo without strain correction. Magn Reson Med. 1999;42(2):393‐403. 10440965 10.1002/(sici)1522-2594(199908)42:2<393::aid-mrm22>3.0.co;2-f

[16] Stoeck CT , von Deuster C , Fleischmann T , Lipiski M , Cesarovic N , Kozerke S . Direct comparison of in vivo versus postmortem second‐order motion‐compensated cardiac diffusion tensor imaging. Magn Reson Med. 2018;79(4):2265‐2276. doi:10.1002/mrm.26871 28833410

[17] Liao R , Podesser BK , Lim CC . The continuing evolution of the Langendorff and ejecting murine heart: New advances in cardiac phenotyping. Am J Physiol Heart Circ Physiol. 2012;303(2):H156‐H167. doi:10.1152/ajpheart.00333.2012 22636675 PMC3404701

[18] Williams DS , Grandis DJ , Zhang W , Koretsky AP . Magnetic resonance imaging of perfusion in the isolated rat heart using spin inversion of arterial water. Magn Reson Med. 1993;30(3):361‐365. doi:10.1002/mrm.1910300314 8412609

[19] Schuster A , Grünwald I , Chiribiri A , et al. An isolated perfused pig heart model for the development, validation and translation of novel cardiovascular magnetic resonance techniques. J Cardiovasc Magn Reson. 2010;12:53. doi:10.1186/1532-429X-12-53 20849589 PMC2950014

[20] Hales PW , Schneider JE , Burton RAB , Wright BJ , Bollensdorff C , Kohl P . Histo‐anatomical structure of the living isolated rat heart in two contraction states assessed by diffusion tensor MRI. Prog Biophys Mol Biol. 2012;110(2‐3):319‐330. doi:10.1016/j.pbiomolbio.2012.07.014 23043978 PMC3526796

[21] Scott AD , Ferreira PFADC , Nielles‐Vallespin S , et al. Optimal diffusion weighting for in vivo cardiac diffusion tensor imaging. Magn Reson Med. 2014;74(2):420‐430. doi:10.1002/mrm.25418 25154715

[22] Scott AD , Nielles‐Vallespin S , Ferreira PF , et al. An in‐vivo comparison of stimulated‐echo and motion compensated spin‐echo sequences for 3 T diffusion tensor cardiovascular magnetic resonance at multiple cardiac phases. J Cardiovasc Magn Reson. 2018;20(1):1. doi:10.1186/s12968-017-0425-8 29298692 PMC5753538

[23] McGill L‐A , Scott AD , Ferreira PF , et al. Heterogeneity of fractional anisotropy and mean diffusivity measurements by in vivo diffusion tensor imaging in normal human hearts. PLoS ONE. 2015;10(7):e0132360. doi:10.1371/journal.pone.0132360 26177211 PMC4503691

[24] McGill LA , Ferreira PF , Scott AD , et al. Relationship between cardiac diffusion tensor imaging parameters and anthropometrics in healthy volunteers. J Cardiovasc Magn Reson. 2016;18:2. doi:10.1186/s12968-015-0215-0 26738482 PMC4704390

[25] Tunnicliffe EM , Scott AD , Ferreira P , et al. Intercentre reproducibility of cardiac apparent diffusion coefficient and fractional anisotropy in healthy volunteers. J Cardiovasc Magn Reson. 2014;16(1):31. doi:10.1186/1532-429X-16-31 24886285 PMC4028111

[26] von Deuster C , Stoeck CT , Genet M , Atkinson D , Kozerke S . Spin echo versus stimulated echo diffusion tensor imaging of the in vivo human heart. Magn Reson Med. 2016;76(3):862‐872. doi:10.1002/mrm.25998 26445426 PMC4989478

[27] Gorodezky M , Scott AD , Ferreira PF , Nielles‐Vallespin S , Pennell DJ , Firmin DN . Diffusion tensor cardiovascular magnetic resonance with a spiral trajectory: An in vivo comparison of echo planar and spiral stimulated echo sequences. Magn Reson Med. 2018;80(2):648‐654. doi:10.1002/mrm.27051 29266435

[28] Tseng W‐YI , Dou J , Reese TG , Wedeen VJ . Imaging myocardial fiber disarray and intramural strain hypokinesis in hypertrophic cardiomyopathy with MRI. J Magn Reson Imaging. 2006;23(1):1‐8. doi:10.1002/jmri.20473 16331592

[29] Wu M‐T , Su M‐YM , Huang Y‐L , et al. Sequential changes of myocardial microstructure in patients postmyocardial infarction by diffusion‐tensor cardiac MR: correlation with left ventricular structure and function. Circ Cardiovasc Imaging. 2009;2(1):32‐40. doi:10.1161/CIRCIMAGING.108.778902 19808562

[30] Casali C , Obadia JF , Canet E , et al. Design of an isolated pig heart preparation for positron emission tomography and magnetic resonance imaging. Invest Radiol. 1997;32(11):713‐720. doi:10.1097/00004424-199711000-00010 9387060

[31] Schuster A , Chiribiri A , Ishida M , et al. Cardiovascular magnetic resonance imaging of isolated perfused pig hearts in a 3T clinical MR scanner. Interv Med Appl Sci. 2012;4(4):186‐192. doi:10.1556/IMAS.4.2012.4.3 24265875 PMC3831784

[32] Kupriyanov VV , Xiang B , Sun J , et al. Three‐dimensional 87Rb NMR imaging and spectroscopy of K+ fluxes in normal and postischemic pig hearts. Magn Reson Med. 2000;44(1):83‐91. doi:10.1002/1522-2594(200007)44:1<83::AID-MRM13>3.0.CO;2-R 10893525

[33] Rose JN , Nielles‐Vallespin S , Ferreira PF , Firmin DN , Scott AD , Doorly DJ . Novel insights into in‐vivo diffusion tensor cardiovascular magnetic resonance using computational modeling and a histology‐based virtual microstructure. Magn Reson Med. 2019;81(4):2759‐2773. doi:10.1002/mrm.27561 30350880 PMC6637383

[34] Chen YF , Said S , Campbell SE , Gerdes AM . A method to collect isolated myocytes and whole tissue from the same heart. Am J Physiol Heart Circ Physiol. 2007;293(3):2006‐2008. doi:10.1152/ajpheart.00479.2007 17513493

[35] Hinken AC , Korte FS , McDonald KS . Porcine cardiac myocyte power output is increased after chronic exercise training. J Appl Physiol. 2006;101(1):40‐46. doi:10.1152/japplphysiol.00798.2005 16565350

[36] Kupriyanov VV , St Jean M , Xiang B , Butler KW , Deslauriers R . Contractile dysfunction caused by normothermic ischaemia and KCl arrest in the isolated pig heart: A 31P NMR study. J Mol Cell Cardiol. 1995;27(8):1715‐1730. doi:10.1016/S0022-2828(95)90854-4 8523433

[37] Kupriyanov VV , Gruwel MLH . Rubidium‐87 magnetic resonance spectroscopy and imaging for analysis of mammalian K+ transport. NMR Biomed. 2005;18(2):111‐124. doi:10.1002/nbm.892 15770627

[38] Kupriyanov VV , Xiang B , Sun J , Jilkina O , Dai G , Deslauriers R . Effects of ischemia on intracellular rubidium in pig and rat hearts 87Rb NMR imaging and spectroscopic study. Magn Reson Med. 2000;44(2):193‐200. doi:10.1002/1522-2594(200008)44:2<193::AID-MRM5>3.0.CO;2-X 10918317

[39] Yang Y , Gruwel MLW , Sun J , Gervai P , Yang X , Kupriyanov VV . Manganese‐enhanced MRI of acute cardiac ischemia and chronic infarction in pig hearts: Kinetic analysis of enhancement development. NMR Biomed. 2009;22(2):165‐173. doi:10.1002/nbm.1297 18756440

[40] Tian G , Shen J , Sun J , et al. Does simultaneous antegrade/retrograde cardioplegia improve myocardial perfusion in the areas at risk? A magnetic resonance perfusion imaging study in isolated pig hearts. J Thorac Cardiovasc Surg. 1998;115(4):913‐924. doi:10.1016/S0022-5223(98)70374-5 9576229

[41] Tian G , Mainwood GW , Biro GP , et al. The effect of high buffer cardioplegia and secondary cardioplegia on cardiac preservation and postischemic functional recovery: A 31P NMR and functional study in Langendorff perfused pig hearts. Can J Physiol Pharmacol. 1991;69(11):1760‐1768. doi:10.1139/y91-260 1804520

[42] Tian G , Biro GP , Butler KW , Xiang B , Vu C , Deslauriers R . The effects of Ca++ on the preservation of myocardial energy and function with University of Wisconsin solution. A(31p) nuclear magnetic resonance study of isolated blood perfused Langendorff pig hearts. J Heart Lung Transplant. 1993;12(1 Pt 1):81‐88. 8443206

[43] Schuster A , Sinclair M , Zarinabad N , et al. A quantitative high resolution voxel‐wise assessment of myocardial blood flow from contrast‐enhanced first‐pass magnetic resonance perfusion imaging: microsphere validation in a magnetic resonance compatible free beating explanted pig heart model. Eur Heart J Cardiovasc Imaging. 2015;16(10):1082‐1092. doi:10.1093/ehjci/jev023 25812572 PMC4570548

[44] Vaillant F , Magat J , Bour P , et al. Magnetic resonance‐compatible model of isolated working heart from large animal for multimodal assessment of cardiac function, electrophysiology, and metabolism. Am J Physiol Heart Circ Physiol. 2016;310(10):H1371‐H1380. doi:10.1152/ajpheart.00825.2015 26968545

[45] Lohezic M , Teh I , Bollensdorff C , et al. Interrogation of living myocardium in multiple static deformation states with diffusion tensor and diffusion spectrum imaging. Prog Biophys Mol Biol. 2014;115(2–3):213‐225. doi:10.1016/j.pbiomolbio.2014.08.002 25117498 PMC4210665

[46] Lohezic M , Bollensdorff C , Korn M , et al. Optimized radiofrequency coil setup for MR examination of living isolated rat hearts in a horizontal 9.4T magnet. Magn Reson Med. 2015;73(6):2398‐2405. doi:10.1002/mrm.25369 25045897

[47] Stoeck CT , von Deuster C , van Gorkum RJH , Kozerke S . Motion and eddy current–induced signal dephasing in in vivo cardiac DTI. Magn Reson Med. 2020;84(1):277‐288. doi:10.1002/mrm.28132 31868257

[48] Brook J , Kim MY , Koutsoftidis S , et al. Development of a pro‐arrhythmic ex vivo intact human and porcine model: cardiac electrophysiological changes associated with cellular uncoupling. Pflugers Arch Eur J Physiol. 2020;472(10):1435‐1446. doi:10.1007/s00424-020-02446-6 32870378 PMC7476990

[49] Peper ES , Leopaldi AM , van Tuijl S , et al. An isolated beating pig heart platform for a comprehensive evaluation of intracardiac blood flow with 4D flow MRI: a feasibility study. Eur Radiol Exp. 2019;3(1):40. doi:10.1186/S41747-019-0114-5 31650367 PMC6813403

[50] Zhong X , Spottiswoode BS , Meyer CH , Kramer CM , Epstein FH . Imaging three‐dimensional myocardial mechanics using navigator‐gated volumetric spiral cine DENSE MRI. Magn Reson Med. 2010;64(4):1089‐1097. doi:10.1002/mrm.22503 20574967 PMC2946451

