# Supplementary material for: Development of a cardiovascular magnetic resonance‐compatible large animal isolated heart model for direct comparison of beating and arrested hearts
Source: NMR Biomed. 2022 Feb 12;35(7):e4692. doi: 10.1002/nbm.4692 (PMC9286060; doi:10.1002/nbm.4692)
Supplement: Supplementary file 1 — Table S1: Perfusate constituents. Table S2: A summary of the success of each of the 10 experiments. Figure S1: Images from the computer aided design files for the MRI compatible perfusion chamber shown in Figure 3. The 3D printed parts are colour coded in A and B. All parts are 3D printed apart from the seals shown in black in A and the transparent acrylic tube shown in A and B. The lid of the chamber is shown in purple, with the barbed connection to inflowing perfusate circuit shown in turquoise and the additional barbed connections for monitoring systems in white. The cannula inserted into the aorta extends out from the turquoise connector into the main chamber (not shown). The structural elements of the support bed are shown in green (A and B) and the same bed is shown with the Voronoi design covering the top and bottom of the structure shown in green in C. The 3D printed lid shown in purple connects to the flange shown in yellow, twisting into place to seal via the rubber o‐rings shown in black. The 3D‐printed chamber support post is shown in red and connects to a machined PTFE post on the flat base holding the chamber. The bottom of the chamber is shown in blue and the barbed effluent ports are visible on the bottom of the chamber. The corresponding STL files used for 3D printing are available on request. Figure S2: Examples of the manual measurements made on the confocal data set. In the original imaging plane (A), the cardiomyocyte length (l) and diameter (d) was measured with the in‐plane rotation (a) of the cardiomyocytes relative to the vertical axis of the image. In the first resliced orientation (B) the rotation of the cardiomyocytes through the image plane was measured (e). In the second resliced orientation (C), the width (w), parallel to the original imaging slice orientation and height (h) perpendicular to the original imaging plane were measured. Figure S3: Results of the measurements performed on the confocal data in the original imaging plane (A‐C [file NBM-35-0-s003.docx]

Supplementary material


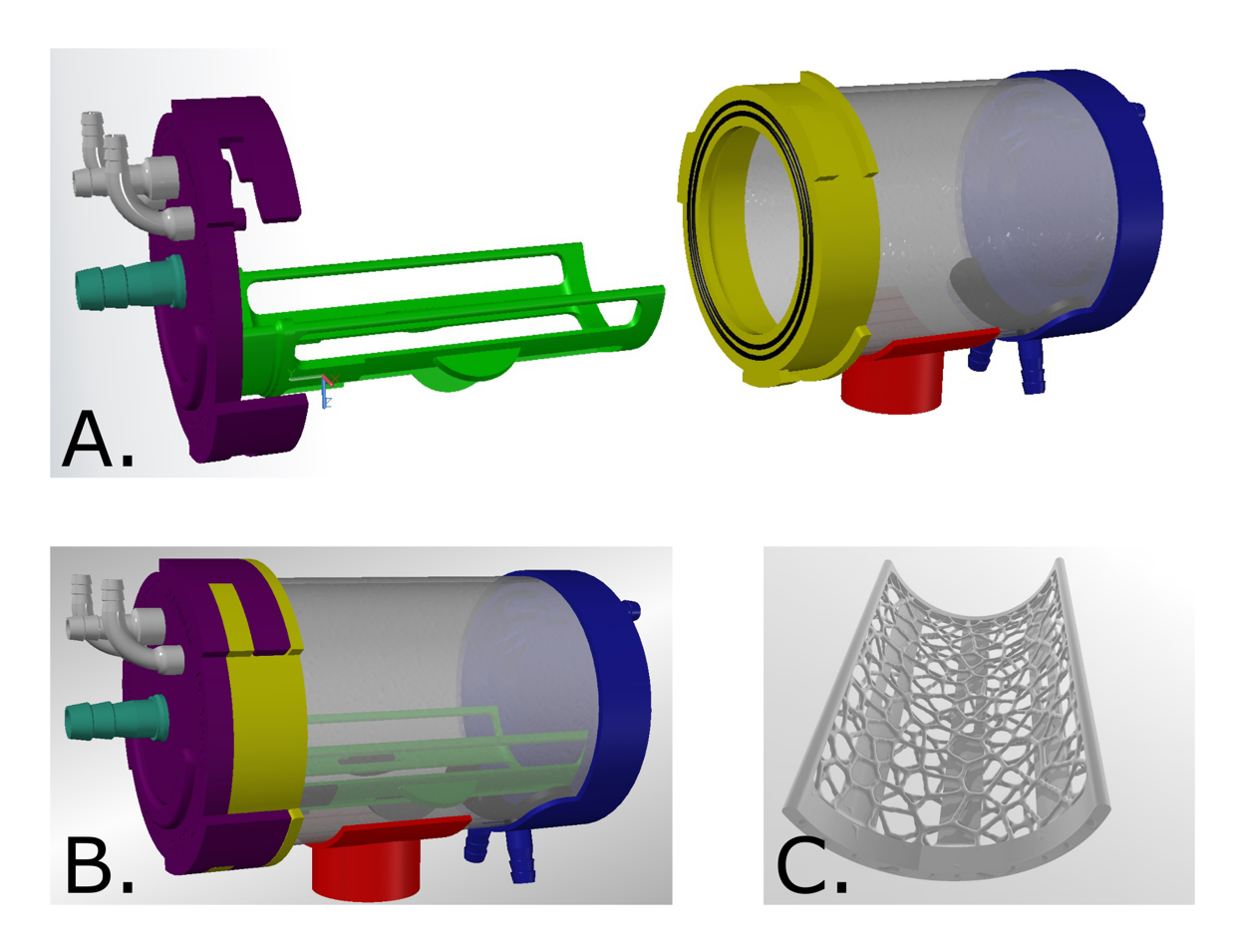


*Supplementary figure 1: Images from the computer aided design files for the MRI compatible perfusion chamber shown in figure 3. The 3D printed parts are colour coded in A and B. All parts are 3D printed apart from the seals shown in black in A and the transparent acrylic tube shown in A and B. The lid of the chamber is shown in purple, with the barbed connection to inflowing perfusate circuit shown in turquoise and the additional barbed connections for monitoring systems in white. The cannula inserted into the aorta extends out from the turquoise connector into the main chamber (not shown). The structural elements of the support bed are shown in green (A and B) and the same bed is shown with the Voronoi design covering the top and bottom of the structure shown in green in C. The 3D printed lid shown in purple connects to the flange shown in yellow, twisting into place to seal via the rubber o-rings shown in black. The 3D-printed chamber support post is shown in red and connects to a machined PTFE post on the flat base holding the chamber. The bottom of the chamber is shown in blue and the barbed effluent ports are visible on the bottom of the chamber. The corresponding STL files used for 3D printing are available on request.*

**Supplementary table 1: Perfusate constituents**

|  | Beating heart “normal” | Slack arrest | Contracted arrest |
| --- | --- | --- | --- |
| LiCl (mmol L^-1^) | 0 | 0 | 125 |
| NaCl (mmol L^-1^) | 119 | 105 | 0 |
| KCl (mmol L^-1^) | 5 | 25 | 5 |
| MgCl_2_ (mmol L^-1^) | 2 | 2 | 2 |
| CaCl_2_ (mmol L^-1^) | 2 | 0 | 2 |
| HEPES (mmol L^-1^) | 25 | 25 | 25 |
| Glucose (mmol L^-1^) | 11 | 11 | 11 |
| Dextran (mmol L^-1^) | 0.596 | 0.596 | 0.596 |
| Insulin (units L^-1^) | 2 | 2 | 2 |
| Heparin (units L^-1^) | 400 | 400 | 400 |
| Approximate volume used per heart (L) | 10 | 5 | 5 |

pH was adjusted to 7.4 using NaOH and HCl.

**Supplementary table 2: A summary of the success of each of the 10 experiments**

| Experiment day/heart number | Regular sustained contraction achieved? | Cold ischemic time (HH:MM) | Time from initial contraction to cease pacing  (HH:MM) | Details |
| --- | --- | --- | --- | --- |
| 1/1 | No | 03:30 |  | The heart hung vertically from the aortic cannula and the LV was un-vented. |
| 1/2 | No | 04:00 |  |  |
| 2/3 | Yes | 03:30 | 02:19 | Used horizonal custom chamber. Stable contraction achieved with pacing. No blood added to the perfusate, chamber filled with perfusate. The heart was unvented but perfusion pressure was low (~15mmHg) and the heart was allowed to warm slowly. Unable to get reliable ECG for MR scanner triggering. |
| 2/4 | No | 05:00 |  | Experimental setup similar to heart #3, with long cold ischemic times. The heart was slowly warmed, but defibrillation was postponed until the heart was ~20˚C. The atria responded to pacing, but the ventricles did not and subsequent defibrillation led to ventricular asystole. |
| 3/5 | No | 05:40 |  | Experimental setup as for heart #4, with an additional heat exchanger at the scanner bed. The chamber was actively drained as filling the chamber with perfusate was found to be unnecessary. The LV was unvented. Ventricular contraction was maintained for ~10minutes, then fibrillation which was unresponsive to shocks. The atria continued to contract spontaneously until the perfusion was stopped. |
| 3/6 | Yes | 03:40 | 01:38 | Experimental setup as for heart #5. The heart was initially transferred from the transport container into clean cardioplegia, allowing time to assess the heart and plan the connection. The LV was vented at the time of connecting the perfusion circuit. After perfusion commenced, the temperature and pressure were quickly increased to physiological levels and the heart began to contract. Once regular paced contraction was established, the perfusate was recirculated and the washed blood was added. We were unable to obtain a reliable ECG signal from the heart, but real time images were acquired in the chamber. The perfusate was exchanged for the contracture formulation, which resulted in arrest without fibrillation within 25minutes of changing perfusate. |
| 4/7 | Yes | 03:15 | 01:41 | Experimental setup as for heart #6, except a syringe of perfusate was gently used to top up the fluid within the aorta to ensure that the aorta did not empty and allow air to enter the coronary arteries. After the perfusion commenced the temperature and pressure were increased rapidly to 50-75mmHg and 38˚C and blood was added. The established beating heart was too swollen to fit through the opening to the chamber. A small flexible surface coil was placed onto the heart beating in a tray on the bed of the scanner and images were acquired. Triggering was achieved via the pacing signal. |
| 4/8 | Yes | 03:30 | 01:17 | Experimental setup as for heart #7. The beating, paced heart perfused with perfusate and washed red blood cells was imaged in the chamber using the pacing signal for triggering. The heart stopped contracting after the initial cine scans. Upon removal from the scanner there was a relatively normal electrical signal, but no contraction. An investigation demonstrated that the heart was twisted at the aorta, causing a coronary occlusion. Once the twisting was rectified the heart resumed regular paced contraction. Imaging was performed in a tray with the flexible coil lying on top of the heart. DT-CMR data was acquired in the most and least contracted cardiac phases in the beating heart. T1 and T2 mapping were performed, before the triggering failed. The pacing was switched off and the perfusate was changed for the contracture formulation. However, DT-CMR data demonstrated sporadic motion-induced signal loss. On removal of the heart from the chamber it was still beating sporadically. 50g of LiCl was added to the perfusate, which induced visible arrest and DT-CMR was repeated. |
| 5/9 | Yes | 05:25 | 01:33 | Experimental setup as for heart #8. The heart was established, paced and perfused with perfusate containing red blood cells. The heart was imaged within the chamber using the pacing signal for triggering. Detection of the triggering during the retrospectively gated bSSFP cine acquisitions failed due to gradient interference on the detected triggering signal. Cine data was successfully acquired using prospectively gated spoiled gradient echo imaging. DT-CMR was acquired from the beating heart in the most and least contracted cardiac phases. T1 and T2 mapping were acquired. The perfusate was switched for the slack formulation and real-time imaging was used to confirm arrest. T1 and T2 mapping and DT-CMR were performed. The heart was removed from the chamber and a block was cut for histology. |
| 5/10 | Yes | 04:30 | 01:17 | Experimental setup as for heart #8. The heart was established, paced and perfused with blood containing perfusate. The heart was imaged within the chamber using the pacing signal for triggering. Cine data was successfully acquired with bSSFP sequence. T1 mapping, T2 mapping, DT-CMR was performed in the most and least contracted cardiac phases. Pacing was stopped and the perfusate was switched for the slack formulation. Real-time imaging was used to establish that arrest was complete, before repeating DT-CMR, T1 and T2 mapping. The perfusate was then switched for the contracture formulation and real-time images were used to monitor the state of the heart. DT-CMR, T1 and T2 mapping were repeated. The heart was removed from the scanner and the histology protocol was followed. |

## CMR imaging protocols

### Cine imaging

Balanced steady state free-precession (bSSFP) cine images were acquired in long and short axis planes based on clinical CMR protocols. Acquisitions were retrospectively gated, with 25 reconstructed frames per cardiac cycle. Field of view: 284 x 380 mm^2^ with 8 mm slice thickness, acquisition matrix 224x168 (frequency x phase encode). TE=1.14ms, TR=5.4ms, flip angle 36˚. GRAPPA acceleration factor 2 and bandwidth per pixel 1490Hz.

### T1 mapping

Modified Look Locker Imaging (MOLLI) was performed with a 5(3)3 protocol, minimum TI 113ms, increment 80ms. Image acquisition was triggered to end diastole and used a bSSFP readout with TE 1.1ms, TR 3.7ms, flip angle 20˚. GRAPPA factor 2, bandwidth per pixel 1085Hz. Field of view 360 x 306mm^2^, slice thickness 6mm, acquisition matrix 256 x 144.

### T2 mapping

T2 prepared images were acquired using a standard product sequence with T2 prep times of 0ms, 30ms, 50ms and 75ms. Image acquisition was triggered to end diastole and used a bSSFP readout with TE=1.3ms, TR=3.7 ms, flip angle 35˚. Field of view 360 x 285mm^2^, slice thickness 6mm, acquisition matrix 192 x 130. Bandwdith per pixel 1370Hz, GRAPPA acceleration factor 2.

### STEAM DT-CMR

Monopolar STEAM-EPI data were acquired with an in-plane spatial resolution of 2x2mm^2^, reconstructed to 1x1mm^2^, with a 6mm slice thickness and a FOV=305x114mm^2^ (read x phase), bandwidth =1960 Hz/pixel, b_main_=500smm^-2^ with 10 averages, b_ref_=150smm^-2^ with 2 averages, each acquired with 6 diffusion encoding directions. The sequence was triggered to alternate cardiac cycles, giving a TR=2 cardiac cycles and a diffusion time, ∆=1 cardiac cycle. Data were acquired with sensitivity encoding (SENSE) factor 2 and TE=31ms with no partial Fourier.

## Histological analysis

## Confocal data

Images were processed in Fiji (Fiji Is Just ImageJ, Version 2.1.0/1.53C)^1^. Manual measurements of cardiomyocyte dimensions (length, width and height) and orientation were performed as shown in supplementary figure 2. In the image acquisition plane (supplementary figure 2.A), the cardiomyocyte length, width and angle relative to the longest dimension of the image were measured. Measurements were performed in 10 slices equally spaced throughout the imaged volume. In each slice, 10 measurements were performed at regularly spaced points on a grid, using a random offset of the grid in each slice.

The confocal volume was then resliced to provide two perpendicular volumes. Supplementary figure 2.B shows an example slice from the first of these resliced volumes. In this plane, the rotation of the cardiomyocytes relative to the image plane was measured. A total of 114 measurements were made in 42 slices from this first resliced volume.

Supplementary figure 2.C shows an example slice from the volume resliced to show the cardiomyocytes in cross-section. Measurements of both the cardiomyocyte width (parallel to the original image plane) and height were made. Measurements were made in a total of 167 locations in 45 slices.

Supplementary figure 3 shows histograms of the measurements described above. The measurements performed in the original imaging plane are shown in the top row (A-C) and the images on the resliced data are shown in D and E. The mean orientation in both cases is close to zero (-4.4˚ in plane and -5.4˚ in the resliced data) suggesting that the imaging plane was well aligned with the cardiomyocyte orientation. The mean length of the cardiomyocytes was 164.7µm, although the distribution appears skewed and the median value is 156.5µm. The mean diameter measured in the imaging plane was 23.5µm, which is close to the mean diameter of the cardiomyocytes measured in cross section of 24.5µm. The cardiomyocytes had a smaller height that their width, with a mean height of 17.4µm. The mean±standard deviation of the aspect ratio of the cardiomyocytes in cross section (width/height) was 1.49±0.25.


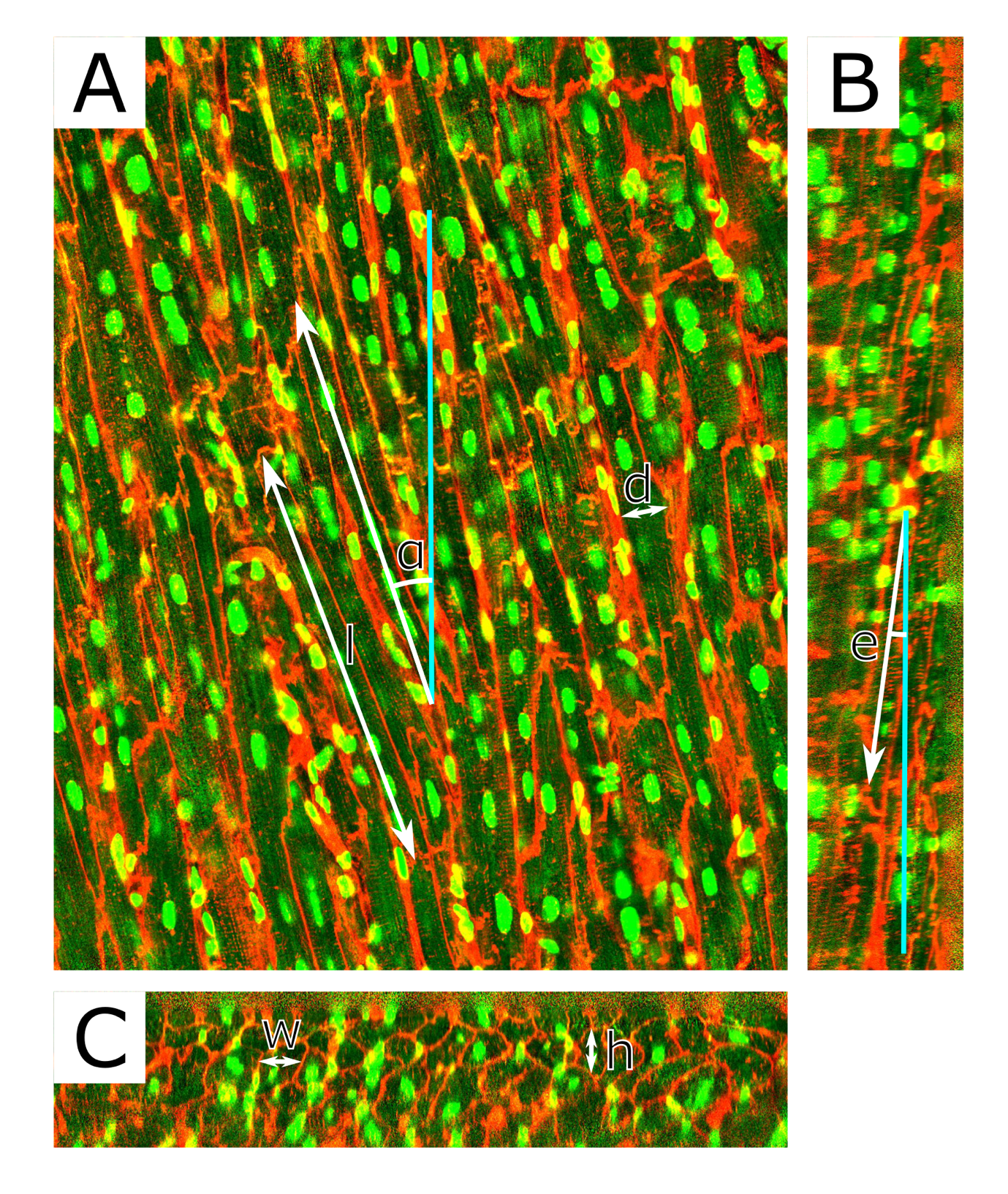


*Supplementary figure 2: Examples of the manual measurements made on the confocal data set. In the original imaging plane (A), the cardiomyocyte length (l) and diameter (d) was measured with the in-plane rotation (a) of the cardiomyocytes relative to the vertical axis of the image. In the first resliced orientation (B) the rotation of the cardiomyocytes through the image plane was measured (e). In the second resliced orientation (C), the width (w), parallel to the original imaging slice orientation and height (h) perpendicular to the original imaging plane were measured.*


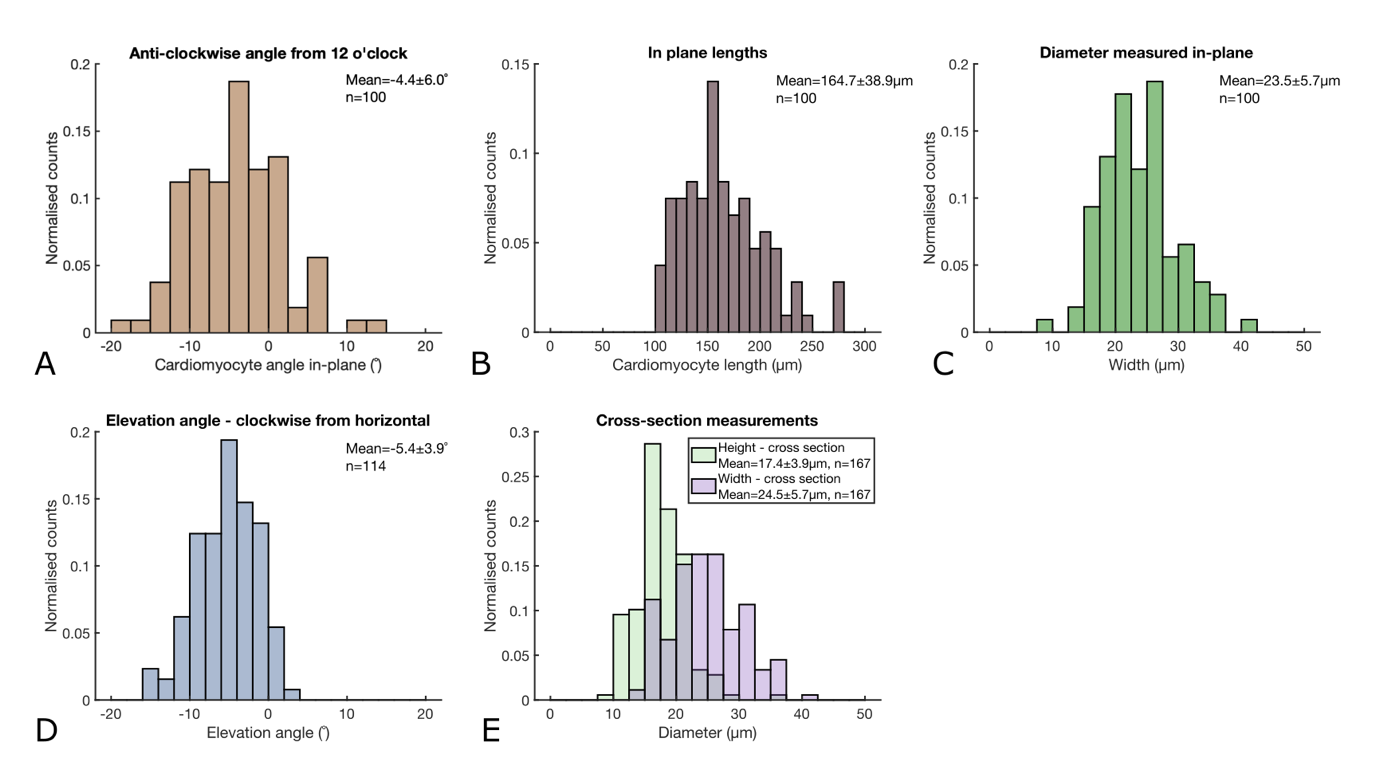


*Supplementary figure 3: Results of the measurements performed on the confocal data in the original imaging plane (A-C) and in planes created by reslicing the data in perpendicular orientations (D and E). The angle in A is defined in supplementary figure 1.A as “a”. The length shown in B and the diameter shown in C and defined in supplementary figure 1.A as “l” and “d” respectively. The elevation angle shown D is defined in supplementary figure 1.B as “e” and the height and width shown in E are defined in supplementary figure 1.C as “w” and “h” respectively.*

## Brightfield imaging

Images at a magnification on 1.25x were extracted from the multi-resolution ndpi files. These images were registered with the “Register_Virtual_Stack_MT” API in ImageJ (<https://javadoc.scijava.org/Fiji/register_virtual_stack/Register_Virtual_Stack_MT.html>), using the rigid option for the features model and moving least squares for the registration. Residual rotation of the slices after registration was corrected in Matlab using an automated routine to align the bottom edge of the slice with the horizontal axis of the image. The registered images are shown in supplementary movie S5.

The images show the cardiomyocytes in cross-section and, therefore, the sheetlet orientation, in the mesocardial region. A rectangular region was selected on the first image that lay within the mesocardium in all slices. Structure tensor analysis of this region in all slices was performed with the ImageJ plugin “OrientationJ” (<http://bigwww.epfl.ch/demo/orientation/#dist>)^2^ using the cubic spline gradient option and $\sigma=8$. Supplementary figure 4, shows the analysed region and the orientations extracted from the structure tensor analysis in an example slice. The resultant angles were rotated to match the definition of E2A in the DTCMR data.

Supplementary figure 5 shows the results of the structure tensor analysis. The peak sheetlet angle was at +62.5˚ and the median absolute sheetlet angle is 59.9˚.


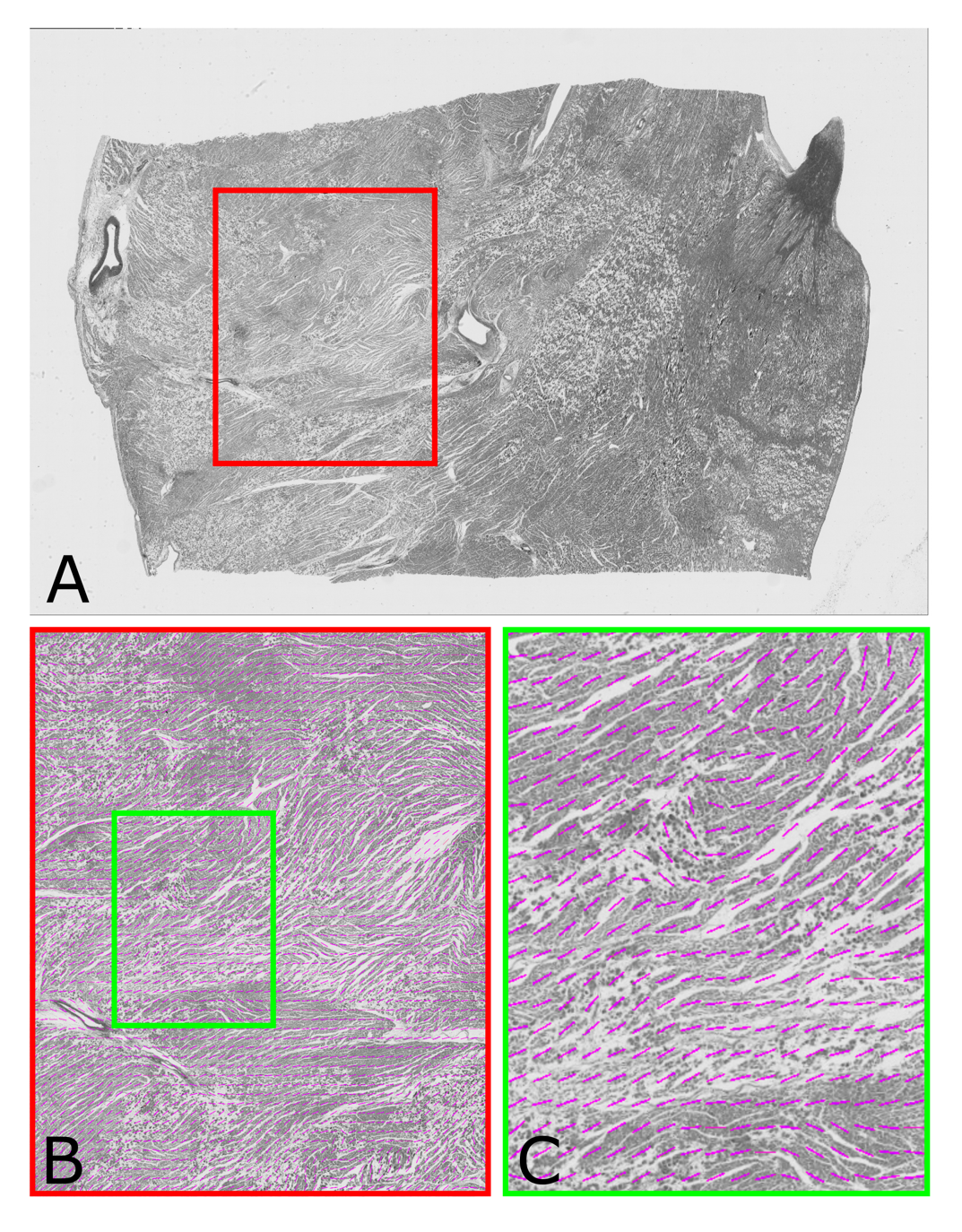


*Supplementary figure 4: Structure tensor analysis applied to an example slice. The images are converted to grayscale (A) and a region is selected that shows the cardiomyocytes in cross section in all slices (red region in A). The selected region is shown in B with the sheetlet orientation extracted using the structure tensor analysis superimposed in magenta. A zoomed in region of the image in B (highlighted in green) in shown in C. The magenta lines are seen to align with the sheetlet orientation.*

*Supplementary figure 5: The results of the structure tensor analysis applied to the brightfield microscopy data presented as a histogram. The histograms from the individual slices are shown in blue and the mean over all slices is shown in red. The sheetlet orientation plotted here is defined as the angle from the vertical orientation in the images, with a clockwise angle shown as positive. Assuming that the local wall tangent is vertical in the images, this definition matches that of the E2A used in DTCMR.*

## References

1. Schindelin J, Arganda-Carreras I, Frise E, et al. Fiji: An open-source platform for biological-image analysis. *Nat Methods*. 2012;9(7):676-682. doi:10.1038/nmeth.2019

2. Rezakhaniha R, Agianniotis A, Schrauwen JTC, et al. Experimental investigation of collagen waviness and orientation in the arterial adventitia using confocal laser scanning microscopy. *Biomech Model Mechanobiol*. 2012;11(3-4):461-473. doi:10.1007/s10237-011-0325-z
